# Low folate status and related polymorphisms are associated with lower sperm quality in healthy males: The Lifestyle and Environmental Determinants of Seminogram and Other Male Fertility-Related Parameters (Led-Fertyl) cross-sectional study

**DOI:** 10.1016/j.ajcnut.2026.101385

**Published:** 2026-06-06

**Authors:** Ailende Eigbefoh-Addeh, Albert Salas-Huetos, Carla Ramos-Rodríguez, Luis A Santos-Calderón, Rashmi Agarwal, Jordi Salas-Salvadó, Nancy Babio, Adrian McCann, Per M Ueland, Michelle M Murphy

**Affiliations:** 1 Unit of Preventive Medicine and Biostatistics, Faculty of Medicine and Health Sciences, Universitat Rovira i Virgili, Reus, Spain; 2 Institut de Recerca Biomèdica Catalunya Sud (IRBCatSud), Reus, Spain; 3 Research Group, Alimentació, Nutrició, Desenvolupament i Salut Mental (ANUT-DSM); 4 Institute of Health Carlos III, Centro de Investigación Biomédica en Red Fisiopatología de la Obesidad y la Nutrición (CIBEROBN), Madrid, Spain; 5 Department of Nutrition, Harvard T.H. Chan School of Public Health, Harvard University, Boston, MA, United States; 6 Department of Biochemistry and Biotechnology, Unit of Human Nutrition, Faculty of Medicine and Health Sciences, Universitat Rovira i Virgili, Reus, Spain; 7 Bevital A/S, Bergen, Norway

**Keywords:** folate, homocysteine, polymorphism, sperm quality, vitamin B_12_

## Abstract

**Background:**

Folate-mediated one-carbon metabolism is essential for deoxyribonucleic acid synthesis, methylation, and genomic integrity during spermatogenesis.

**Objectives:**

To study the associations between the status of folate, cobalamin, homocysteine, single nucleotide polymorphisms (SNPs) affecting folate-mediated one-carbon metabolism, and sperm quality.

**Methods:**

In this cross-sectional study, 197 healthy males (18‒40 y) were recruited between 2021 and 2023. Plasma folate, red blood cell folate (RBCF), plasma cobalamin, total homocysteine, and SNPs affecting folate and cobalamin status were determined. Sperm quality parameters were assessed by World Health Organization 2010 criteria. Associations between B-vitamin status, genetic polymorphisms, and sperm quality parameters were assessed using multivariable linear and logistic regression analysis.

**Results:**

Median (25th and 75th percentiles) plasma folate was 14.2 (11.0, 17.8) nmol/L, and RBCF was 678.2 (582.3, 785.0) nmol/L. Deficiency, plasma folate <7 nmol/L and RBCF (<340 nmol/L), occurred in 4.6% [95% confidence interval (CI): 2.4%, 8.5%] and 2.6% (95% CI: 1.1%, 5.8%) of participants, respectively. Lowest compared with highest tertile plasma folate (<11.9 nmol/L compared with ≥17.0 nmol/L) and RBCF (<625.0 nmol/L compared with ≥742.1 nmo/L) were associated with lower sperm count [B = ‒0.428, standard error (SE): 0.196 and B = ‒0.459, SE: 0.183, respectively] and higher odds of altered seminogram [odds ratio: (OR): 3.4; 95% CI: 1.4, 8.2 and OR: 2.3; 95% CI: 1.0, 5.3, respectively]. Median (25th and 75th percentiles) plasma cobalamin was 239.1 (203.0, 282.3) pmol/L and plasma total homocysteine was 9.6 (7.8, 11.5) μmol/L. Cobalamin deficiency (<148 pmol/L) and plasma total homocysteine >15 μmol/L were observed in 1.5% (95% CI: 0.5%, 4.4%) and 5.6% (95% CI: 3.1%, 9.7%) of participants, respectively. Neither was associated with sperm quality parameters. The *MTHFD1* 1958AA compared with GG genotype was associated with reduced progressive motility (B = ‒7.7%, SE: 3.8), and higher odds of altered seminogram (OR: 4.4; 95% CI: 1.4, 13.7).

**Conclusions:**

Low folate status is negatively associated with sperm quality. The *MTHFD1* 1958AA genotype may be negatively associated with sperm quality.

## Introduction

Folate (vitamin B_9_) mediated one-carbon metabolism is critical for DNA synthesis, replication, and repair in rapidly dividing germ cells [1]. Folate and cobalamin (vitamin B_12_) play interdependent roles in methyl donor supply to epigenetic reactions via homocysteine remethylation to methionine [2]. Regarding their roles in reproduction, research has largely focused on female folate status. Low maternal folate status has been associated with first-trimester miscarriage [3], and the critical role of maternal folate status in early fetal development and neural-tube closure has motivated mandatory folic acid fortification policies [4] and the inclusion of folic acid supplementation in prenatal clinical guidelines globally [5].

Pregnancy complications or adverse pregnancy outcomes [[6], [7], [8], [9], [10], [11]] have been reported in mothers carrying variant genotypes for single nucleotide polymorphisms (SNPs) affecting folate and cobalamin metabolism. These include methylenetetrahydrofolate reductase (*MTHFR*) 677C>T (rs1801133) [12], reduced folate carrier [solute carrier family 19A, member 1 (*SLC19A1*)] 80G>A (rs1051266) [13], methylenetetrahydrofolate dehydrogenase-5,10-methylenetetrahydrofolate cyclohydrolase-10-formyltetrahydrofolate synthetase (*MTHFD1*) 1958G>A (rs2236225) [14], methionine synthase reductase (*MTRR*) 524C>T (rs1532268) [15], *MTRR* 66A>G (rs1801394) [16], and transcobalamin II (*TCII*) 776C>G (rs1801198) [17]. However, few studies have investigated whether the paternal genetic component is associated with adverse pregnancy outcomes.

Folate-mediated one-carbon metabolism is closely linked to spermatogenesis, a tightly regulated process encompassing mitotic proliferation, meiotic division, and spermiogenesis, and participates in fundamental mechanisms in genomic integrity and sperm function maintenance [1,2,18]. In males attending fertility clinics, folate deficiency has been associated with altered sperm quality parameters and increased sperm DNA damage [19,20]. Cobalamin status has been proposed to influence the maturation of human spermatozoa [21] and has been positively associated with sperm quality [22,23]. Homocysteine concentration in both ejaculated sperm and follicular fluid has been negatively associated with embryo quality [24].

Given the central role of one-carbon metabolism in cell division and epigenetic processes, low folate and/or cobalamin status could be associated with impaired spermatogenesis and sperm quality. Evidence from non-clinical settings is limited. We hypothesize that folate and cobalamin status are positively associated with sperm quality parameters. The aim of the study is to investigate the association between folate, cobalamin, and homocysteine status biomarkers, polymorphisms affecting these or their roles, and sperm quality parameters, in healthy adult males of reproductive age unexposed to mandatory fortification with folic acid.

## Methods

### Participants

The study design, population, and recruitment procedures have been described in detail previously [25]. The first 200 participants in the lifestyle and environmental determinants of seminogram and other male fertility-related parameters (Led-Fertyl) study, recruited between February 2021 and April 2023, were studied. Healthy male volunteers aged 18‒40 y, and free of severe chronic diseases, reproductive issues, infectious diseases, recent weight loss (≥5 kg), illegal substance abuse, or any condition affecting adherence to the study protocol were eligible to participate. Participants were recruited following a publicity campaign via social networks and notice boards, from the University population, extended contacts, and the general public in local cities in Southern Catalonia (Spain).

### Ethical approval

The study was approved by the Institut d’Investigació Sanitària Pere Virgili ethical committee (reference: CEIM: 181/2019), and the study was conducted in accordance with the Declaration of Helsinki for Medical Research involving human subjects. All participants provided their signed informed consent.

### Sociodemographic and lifestyle

Participants attended in-person check-ups at the Hospital Universitari Sant Joan de Reus, where weight, height, waist circumference, and blood pressure were measured, and fasting blood and semen samples were collected. Lifestyle and sociodemographic data, including smoking habits, physical activity, age, education level, and income, were recorded via online questionnaires. Data on supplement or vitamin use were not collected.

### Blood samples

Overnight fasting blood samples were collected into EDTA-K_2_ vacutainers. All samples were immediately kept in coolers with cooling packs and processed <2 h after blood draw. Aliquots of plasma and whole blood, diluted 1/10 with 1% ascorbic acid solution [for red blood cell folate (RBCF) measurements], were subsequently stored at ‒80°C until further analysis. Leukocytes were isolated from the blood samples and DNA extracted from these using the QIAGEN DNeasy Blood and Tissue Kit (QIAGEN). Fasting concentrations of plasma and RBCF (microbiological assay, *Lactobacillus casei*) [26], plasma cobalamin (vitamin B_12_) (microbiological assay, *Lactobacillus leichmannii*) [27], and plasma total homocysteine (tHcy) (Liquid Chromatography-Tandem Mass Spectrometry; LC-MS/MS) [28] were determined by Bevital (www.bevital.no). Deficiencies were defined as plasma folate <7 nmol/L [29], RBCF <340 nmol/L [30], and plasma vitamin B_12_ <148 pmol/L [31]. Elevated homocysteine was defined as tHcy >15 μmol/L [31]. In routine clinical settings, plasma or serum folate concentration is the widely used measurement of folate status. It is sensitive to recent dietary folate or folic acid intake. On the contrary, RBCF reflects folate uptake during erythropoiesis and folate status over the 120 d prior to blood draw. This measurement might more accurately reflect folate status during spermatogenesis, which occurs ≥64‒74 d before sample collection.

*MTHFR* 677C>T (rs1801133), *SLC19A1* 80G>A (rs1051266), *MTHFD1* 1958G>A (rs2236225), *MTRR* 66A>G (rs1801394), *MTRR* 524C>T (rs1532268), and *TCII* 776C>G (rs1801198) genotypes were determined at the Spanish National Genotyping Center (Centro Nacional de Genotipado-Fundación Pública Galega de Medicina Xenómica; CEGEN-FPGMX) by the MassARRAY-CPM System and iPLEX Gold chemistry (Agena Bioscience) as described previously [32].

### Semen quality parameters

All semen analyses were performed on fresh samples within 60 min of collection. Participants collected semen samples in sterile polypropylene containers after a minimum of 3 d of abstinence from sexual activity. Semen processing was performed post-liquefaction (30 min at 37°C), according to the WHO guidelines [33]. Detailed procedures have been described previously by our group [25]. Reference lower limits (RLL) for semen parameters were defined according to WHO 2010 criteria [34]. Microscopic characteristics, including sperm count (RLL = 39 million spermatozoa per ejaculate), sperm concentration (RLL = 15 million spermatozoa per milliliter), total motility (RLL = 40%), progressive motility (RLL = 32%), vitality (RLL = 58%), and morphology (RLL = 4% normal forms), were analyzed using a phase-contrast microscope (CX43 Olympus) coupled with a validated computer-assisted sperm analysis system (Sperm Class Analyzer; SCA, Microptic) [35].

### Statistical analysis

The sample size for the Led-Fertyl cohort was determined using a precision-based approach. Assuming a prevalence of progressive sperm motility of 43.5%, a margin of error of 7%, and a 95% confidence level, the minimum required sample size was calculated to be 192 participants. Continuous variables are reported as median (25th, 75th percentiles) and categorical variables are reported as percentage [95% confidence interval (CI)]. Normal distribution and homogeneity of variances were evaluated using Kolmogorov–Smirnov and Levene’s test, respectively. Total sperm count, sperm concentration, sperm vitality, total motility, and normal sperm forms had skewed distributions and were natural log transformed to approach a normal distribution. Between-group comparisons of quantitative variables were performed using the Kruskal–Wallis test. Post hoc Bonferroni correction for multiple comparisons was applied when necessary. Differences between categorical variables were assessed using the Pearson χ^2^-test or Fisher’s exact test, as appropriate. Spearman correlation coefficients were calculated to assess relationships between B-vitamin status biomarkers and sperm quality parameters.

Missing data were handled using a complete-case approach in which analyses were performed using all available data for each model, and no imputation was applied. The vitamin and metabolite status data were categorized into tertiles to allow for the assessment of potential nonlinear associations, to reduce the influence of skewed distributions and extreme values, and to facilitate interpretability of results in this study population, where no strong a-priori assumption of linearity was made. The data-driven tertile categories were cross-checked against cut-offs for males aged <50 y from a randomly selected population sample from the same region, analyzed using the same assays and laboratory procedures [36]. This confirmed that the categories resulting from the applied stratification were generally on par with low and high vitamin status observed in the population.

Multivariable linear regression models were fitted to assess the association between low compared with mid and high tertiles of plasma folate, RBCF, plasma vitamin B_12_, plasma tHcy, and sperm quality parameters (total sperm count, sperm concentration, sperm vitality, total motility, progressive motility, and normal sperm forms). For plasma folate, RBCF, and plasma vitamin B_12_, the highest tertiles were used as the reference group, whereas for plasma tHcy, the lowest tertile was used as the reference. Nonstandardized regression coefficients [B (SE)] are reported for the crude and adjusted models. Model 1 was adjusted for plasma folate (in the plasma vitamin B_12_ model), plasma vitamin B_12_ (in the plasma folate and RBCF models), age, BMI (in kg/m^2^), education, smoking status, physical activity, sexual activity abstinence, and plasma creatinine. In the case of the model testing plasma tHcy as an independent variable, plasma folate and plasma vitamin B_12_ were excluded from the models. Model 2 was further adjusted for relevant genetic polymorphisms, depending on the vitamin of interest. Specifically, plasma folate and RBCF model 2 were additionally adjusted for *MTHFR* 677CT compared with CC, *MTHFR* 677TT compared with CC, *SLC19A1* 80GA compared with GG, *SLC19A1* 80AA compared with GG, *MTHFD1* 1958GA compared with GG, and *MTHFD1* 1958AA compared with GG. Plasma vitamin B_12_ model 2 was additionally adjusted for *MTRR* 524CT compared with CC, *MTRR* 524TT compared with CC, *MTRR* 66AG compared with AA, *MTRR* 66GG compared with AA, *TCII* 776CG compared with CC, and *TCII* 776GG compared with CC. Plasma tHcy model 2 was additionally adjusted for all 6 SNPs. In addition, multivariable logistic regression models were used to estimate odds ratios (ORs) and the 95% CI for the associations between SNPs and altered seminogram according to WHO 2010 normality thresholds, adjusted for the aforementioned confounders. Gene-nutrient and nutrient-nutrient interactions were tested in the regression models by including the interaction term (variable 1 ∗ variable 2) as an independent variable. Given the evaluation of multiple biomarkers and outcomes, the primary analyses were restricted to a-priori hypotheses and assessed using a conventional significance threshold (*P* < 0.05). For secondary and exploratory analyses, including SNP and correlations, adjustment for multiple testing was performed using Bonferroni correction to reduce the risk of type I error, because these analyses were considered hypothesis-generating. The potential for confounding was assessed using an approach that integrated prior knowledge, informed by directed acyclic graphs (DAG) (Supplemental Figure 1), with descriptive statistics from our cohort. Statistical analyses were performed using SPSS version 29.0 (SPSS Inc.) or R v.4.6.0 (R Foundation for Statistical Computing). Statistical significance was set at *P* < 0.05.

## Results

### Characteristics of study population

A total of 320 individuals were assessed for eligibility to participate; 84 declined participation, and 12 did not meet inclusion criteria. Of the 224 enrolled, 24 dropped out. Finally, 3 azoospermic participants were excluded, as semen quality parameters could not be quantified in the absence of sperm, leaving 197 participants for the final analysis (Supplemental Figure 2). Overall, 40.0% had ≥1 altered sperm quality parameter (Table 1).

**TABLE 1:** Overall baseline characteristics of the study population

Demographic parameters	*n*	Overall
Age (y); median (P25-P75)	197	28.0 (24.0, 32.0)
BMI (kg/m^2^); median (P25-P75)	197	24.4 (22.2, 25.9)
Physical activity (MET min/wk); median (P25-P75)	197	3595 (1768, 5315)
Sexual abstinence (days); median (P25-P75)	197	4.0 (3.0, 5.0)
Smoking status; % (95% CI)		
Non-smoker	136	73.5 (66.7, 79.3)
Current smoker	24	13.0 (8.9, 18.6)
Former smoker	25	13.5 (9.3, 19.2)
Education; % (95% CI)		
High school or less	70	35.5 (29.2, 42.4)
College or higher education	127	64.5 (57.6, 70.8)
Blood parameters		
Creatinine (μmol/L); median (P25-P75)	196	82.2 (75.2, 89.3)
Plasma folate (nmol/L); median (P25-P75)	197	14.2 (11.0, 17.8)
RBCF (nmol/L); median (P25-P75)	196	678.2 (582.3, 785.0)
Plasma vitamin B_12_ (pmol/L); median (P25-P75)	197	239.1 (203.0, 282.3)
Plasma tHcy (μmol/L); median (P25-P75)	197	9.6 (7.8, 11.5)
Plasma folate <7 nmol/L; % (95% CI)	197	4.6 (2.4, 8.5)
RBCF <340 nmol/L; % (95% CI)	196	2.6 (1.1, 5.8)
Plasma vitamin B_12_ <148 pmol/L; % (95% CI)	197	1.5 (0.5, 4.4)
Plasma tHcy >15 μmol/L; % (95% CI)	197	5.6 (3.1, 9.7)
Sperm parameters		
Total sperm count (x10^6^ spz.); median (P25-P75)	197	168.0 (98.0, 285.0)
Sperm concentration (x10^6^ spz./mL); median (P25-P75)	197	49.0 (29.0, 82.0)
Sperm vitality (%); median (P25-P75)	196	81.8 (75.9, 88.5)
Total motility (%); median (P25-P75)	197	61.5 (47.5, 73.4)
Progressive motility (%); median (P25-P75)	197	45.6 (31.0, 56.5)
Normal sperm morphology (%); median (P25-P75)	196	9.3 (5.0, 15.0)
Total sperm count <39 × 10^6^ spz.; % (95% CI)	197	8.1 (5.1, 12.8)
Sperm concentration <15 × 10^6^ spz./mL; % (95% CI)	197	9.6 (6.3, 14.6)
Sperm vitality <58%; % (95% CI)	196	4.6 (2.4, 8.5)
Total motility <40% motile; % (95% CI)	197	11.2 (7.5, 16.3)
Progressive motility <32% motile; % (95% CI)	197	26.9 (21.2, 33.5)
Normal sperm morphology <4% normal; % (95% CI)	196	13.8 (9.6, 19.3)
Altered seminogram^1^; % (95% CI)	195	40.0 (33.4, 47.0)
Polymorphism		
*MTHFR* 677 C>T; % (95% CI)		
CC	86	44.1 (37.3, 51.1)
CT	83	42.6 (35.8, 49.6)
TT	26	13.3 (9.3, 18.8)
*SLC19A1* 80G>A; % (95% CI)		
GG	50	26.2 (20.5, 32.8)
GA	92	48.2 (41.2, 55.2)
AA	49	25.7 (20.0, 32.3)
*MTHFD1* 1958G>A; % (95% CI)		
GG	72	36.9 (30.5, 43.9)
GA	95	48.7 (41.8, 55.7)
AA	28	14.4 (10.1, 20.0)
*MTRR* 524C>T; % (95% CI)		
CC	96	49.2 (42.3, 56.2)
CT	74	37.9 (31.4, 44.9)
TT	25	12.8 (8.8, 18.2)
*MTRR* 66 A>G; % (95% CI)		
AA	56	28.9 (22.9, 35.6)
AG	86	44.3 (37.5, 51.4)
GG	52	26.8 (21.1, 33.4)
*TCII* 776C>G; % (95% CI)		
CC	64	32.8 (26.6, 39.7)
CG	88	45.1 (38.3, 52.1)
GG	43	22.1 (16.8, 28.4)

Continuous variables are reported as median (25th and 75th percentiles) and categorical variables as percentage (95% CI). Given the small number of observations in certain subgroups, CIs should be interpreted with caution.

Abbreviations: BMI, body mass index; CI, confidence interval; MET, metabolic equivalent of task; *MTHFD1,* methylenetetrahydrofolate dehydrogenase-5,10-methylenetetrahydrofolate cyclohydrolase-10-formyltetrahydrofolate synthetase; *MTHFR,* methylenetetrahydrofolate reductase; *MTRR,* methionine transferase reductase; *n*, number of subjects; P25-P75, 25th and 75th percentiles; RBCF, red blood cell folate; *SLC19A1,* solute carrier family 19 (folate transporter) member 1; *TCII,* transcobalamin II; tHcy, fasting total homocysteine; WHO, World Health Organization.

1 Participants who have 1 or more altered sperm quality parameters according to WHO 2010 thresholds.

### B-vitamin status biomarkers and sperm quality

Participant characteristics according to tertiles of B-vitamin status biomarkers are reported in Table 2. Those in the highest tertile of plasma folate concentration had lower BMI and higher physical activity compared to those in the lowest tertile. Smoking prevalence was higher in the lowest RBCF tertile group, compared to the mid and high RBCF tertile groups. Sperm quality parameters according to tertiles of B-vitamin status biomarkers are reported in Table 3. The prevalence of low sperm count (<39 × 10^6^ spermatozoa (spz)), concentration (<15 × 10^6^ spz./mL), total motility (<40% motile), progressive motility (<32% motile), and overall altered semen profiles were lower in the highest tertile compared with the lowest tertile group. Sperm quality parameters did not differ among the RBCF tertiles. The prevalence of low progressive motility (<32% motile) was higher in the highest vitamin B_12_ tertile compared to the lowest tertile. No differences were observed in sperm quality parameters between the different tHcy tertiles (Table 3).

**TABLE 2:** Demographic and plasma B vitamin and tHcy concentrations in the lowest and highest tertiles of status at baseline

Characteristics	Plasma folate (nmol/L)			RBCF (nmol/L)			Plasma vitamin B_12_ (pmol/L)			Plasma tHcy (μmol/L)		
	T1 (≤11.87)	T2 (>11.87‒<17.00)	T3 (≥17.00)	T1 (≤624.95)	T2 (>624.95‒<742.11)	T3 (≥742.11)	T1 (≤213.30)	T2 (>213.30‒<265.90)	T3 (≥265.90)	T1 (≤8.40)	T2 (>8.40‒<10.69)	T3 (≥10.69)
*n*	65	66	66	65	66	65	65	66	66	65	66	66
Demographic parameters												
Age (y); median (P25-P75)	29.0 (24.0, 32.0)	28.5 (24.0, 32.8)	28.0 (24.0, 32.0)	29.0 (25.0, 34.0)	27.0 (22.5, 32.0)	28.0 (24.0, 32.0)	28.0 (25.0, 33.0)	28.0 (23.0, 31.0)	29.0 (23.2, 33.8)	29.0 (24.0, 33.0)	26.0 (22.2, 31.8)	29.0 (26.0, 32.0)
BMI (kg/m^2^); median (P25-P75)	25.3 (23.1, 27.1)	24.5 (21.7, 25.7)	23.8 (21.8, 25.0)^1^	24.5 (22.2, 26.3)	24.5 (21.9, 26.0)	24.0 (22.6, 25.7)	24.7 (22.6, 26.0)	24.4 (22.6, 26.8)	24.0 (21.7, 25.4)	24.1 (22.0, 25.4)	24.0 (21.9, 25.2)	25.3 (22.8, 28.0)
Physical activity (MET min/wk); median (P25-P75)	2615 (1445, 4849)	3359 (1720, 4753)	4267 (3072, 5957)^1^	3040 (1570, 4896)	3950 (1899, 5473)	3639 (2033, 5315)	2741 (1678, 5049)	3693 (2056, 5422)	4212 (1787, 5423)	3864.8 (1986.9, 5876.0)	3755.2 (1889.3, 5312.9)	3026.1 (1701.6, 4770.4)
Sexual abstinence (d); median (P25-P75)	3.0 (3.0, 4.0)	4.0 (3.0, 5.0)	4.0 (3.0, 5.0)	3.0 (3.0, 5.0)	4.0 (3.0, 5.8)	4.0 (3.0, 4.0)	4.0 (3.0, 5.0)	4.0 (3.0, 5.0)	3.0 (3.0, 5.0)	4.0 (3.0, 5.0)	4.0 (3.0, 4.0)	4.0 (3.0, 5.0)
Smoking status; % (95% CI)												
Non-smoker	69.8 (57.6, 79.8)	70.0 (57.5, 80.1)	80.6 (69.1, 88.6)	66.7 (54.4, 77.1)	72.6 (60.4, 82.1)	81.4 (69.6, 89.3)	71.9 (59.2, 81.9)	74.6 (62.7, 83.7)	73.8 (62.0, 83.0)	73.8 (61.6, 83.2)	77.4 (65.6, 86.0)	69.4 (57.0, 79.4)
Current smoker	20.6 (12.5, 32.2)	11.7 (5.8, 22.2)	6.5 (2.5, 15.4)	23.8 (15.0, 35.6)	8.1 (3.5, 17.5)^1^	6.8 (2.7, 16.2)^1^	19.3 (11.1, 31.3)	7.9 (3.4, 17.3)	12.3 (6.4, 22.5)	13.1 (6.8, 23.8)	9.7 (4.5, 19.5)	16.1 (9.0, 27.2)
Former smoker	9.5 (4.4, 19.3)	18.3 (10.6, 29.9)	12.9 (6.7, 23.4)	9.5 (4.4, 19.3)	19.4 (11.4, 30.9)	11.9 (5.9, 22.5)	8.8 (3.8, 18.9)	17.5 (10.0, 28.6)	13.8 (7.5, 24.3)	13.1 (6.8, 23.8)	12.9 (6.7, 23.4)	14.5 (7.8, 25.3)
Education; % (95% CI)												
High school or less	46.2 (34.6, 58.1)	31.8 (21.8, 43.8)	28.8 (19.3, 40.6)	35.4 (24.9, 47.5)	39.4 (28.5, 51.5)	32.3 (22.2, 44.4)	32.3 (22.2, 44.4)	33.3 (23.2, 45.3)	40.9 (29.9, 53.0)	41.5 (30.4, 53.7)	33.3 (23.2, 45.3)	31.8 (21.8, 43.8)
College or higher education	53.8 (41.9, 65.4)	68.2 (56.2, 78.2)	71.2 (59.4, 80.7)	64.6 (52.5, 75.1)	60.6 (48.5, 71.5)	67.7 (55.6, 77.8)	67.7 (55.6, 77.8)	66.7 (54.7, 76.8)	59.1 (47.0, 70.1)	58.5 (46.3, 69.6)	66.7 (54.7, 76.8)	68.2 (56.2, 78.2)
Blood parameters												
Creatinine (μmol/L); median (P25-P75)	83.1 (75.2, 89.3)	83.6 (76.3, 89.3)	80.5 (74.3, 88.4)	83.1 (75.2, 89.3)	81.8 (75.2, 89.7)	80.9 (74.9, 88.6)	79.6 (74.3, 88.4)	82.7 (76.3, 91.1)	83.1 (75.2, 88.4)	80.5 (73.4, 87.5)	81.8 (76.3, 88.2)	85.8 (75.4, 91.7)
Plasma folate (nmol/L); median (P25-P75)	9.8 (7.9, 10.9)	14.1 (13.1, 15.2)^1^	19.9 (17.8, 25.4)^1^^,^^2^	10.5 (8.4, 13.1)	14.8 (12.3, 17.4)^1^	18.1 (14.5, 23.8)^1^^,^^2^	13.7 (10.7, 16.3)	14.3 (11.1, 18.7)	14.2 (11.3, 18.1)	17.0 (13.4, 19.7)	13.6 (11.6, 18.0)^1^	12.2 (8.9, 15.5)^1^
RBCF (nmol/L); median (P25-P75)	557.1 (452.7, 676.9)	677.0 (604.4, 746.8)^1^	803.3 (682.8, 982.7)^1^^,^^2^	503.3 (440.5, 574.0)	678.2 (658.0, 700.4)^1^	887.0 (791.0, 1005.9)^1^^,^^2^	679.0 (537.6, 751.4)	679.3 (608.2, 868.5)	676.8 (590.1, 769.0)	727.7 (655.9, 892.0)	676.2 (596.4, 803.2)^1^	648.9 (481.6, 700.9)^1^
Plasma vitamin B_12_ (pmol/L); median (P25-P75)	232.3 (193.7, 279.3)	229.2 (195.3, 281.7)	250.6 (215.4, 296.8)	229.9 (187.0, 278.8)	246.6 (213.7, 291.3)	239.1 (199.1, 279.6)	191.5 (174.7, 202.0)	238.8 (225.0, 255.5)^1^	311.7 (282.4, 335.9)^1^^,^^2^	264.1 (225.0, 324.5)	239.9 (206.8, 279.1)	218.5 (192.4, 259.5)^1^
Plasma tHcy (μmol/L); median (P25-P75)	10.7 (9.2, 13.3)	9.6 (8.2, 11.4)^1^	8.4 (6.8, 9.8)^1^^,^^2^	10.6 (9.1, 13.4)	9.4 (7.5, 10.9)	8.5 (7.2, 10.3)^1^	10.3 (8.4, 12.1)	10.0 (8.4, 11.1)	8.5 (7.0, 10.2)^1^	7.2 (6.5, 7.8)	9.5 (8.9, 10.1)^1^	12.7 (11.5, 14.3)^1^^,^^2^
Plasma folate <7 nmol/L; % (95% CI)	13.8 (7.5, 24.3)	0.0 (0.0, 5.5)^1^	0.0 (0.0, 5.5)^1^	13.8 (7.5, 24.3)	0.0 (0.0, 5.5)^1^	0.0 (0.0, 5.6)^1^	4.6 (1.6, 12.7)	1.5 (0.3, 8.1)	7.6 (3.3, 16.5)	1.5 (0.3, 8.2)	3.0 (0.8, 10.4)	9.1 (4.2, 18.4)
RBCF <340 nmol/L; % (95% CI)	4.6 (1.6, 12.7)	3.0 (0.8, 10.4)	0.0 (0.0, 5.6)	7.7 (3.3, 16.8)	0.0 (0.0, 5.5)^1^	0.0 (0.0, 5.6)^1^	4.6 (1.6, 12.7)	0.0 (0.0, 5.6)	3.0 (0.8, 10.4)	3.1 (0.8, 10.5)	0.0 (0.0, 5.6)	4.5 (1.6, 12.5)
Plasma vitamin B_12_ <148 pmol/L; % (95% CI)	4.6 (1.6, 12.7)	0.0 (0.0, 5.5)^1^	0.0 (0.0, 5.5)^1^	4.6 (1.6, 12.7)	0.0 (0.0, 5.5)^1^	0.0 (0.0, 5.6)^1^	4.6 (1.6, 12.7)	0.0 (0.0, 5.5)	0.0 (0.0, 5.5)	0.0 (0.0, 5.6)	1.5 (0.3, 8.1)	3.0 (0.8, 10.4)
Plasma tHcy >15 μmol/L; % (95% CI)	10.8 (5.3, 20.6)	6.1 (2.4, 14.6)	0.0 (0.0, 5.5)^1^^,^^2^	12.3 (6.4, 22.5)	1.5 (0.3, 8.1)^1^	3.1 (0.8, 10.5)^1^	10.8 (5.3, 20.6)	1.5 (0.3, 8.1)	4.5 (1.6, 12.5)	0.0 (0.0, 5.6)	0.0 (0.0, 5.5)	16.7 (9.6, 27.4)^1^^,^^2^

Continuous variables are reported as median (25th and 75th percentiles), and categorical variables as percentage (95% CI). *P* value was calculated by the Kruskal–Wallis test for continuous variables and then corrected for Bonferroni correction for multiple comparisons. χ^2^test was used to compare categorical variables.

Abbreviations: BMI, body mass index; CI, confidence interval; MET, metabolic equivalent of task; *n*, number of subjects; P25-P75, 25th and 75th percentiles; RBCF, red blood cell folate; T, tertile; tHcy, fasting total homocysteine.

1 *P* < 0.05 for comparison of specific T compared with T1.

2 *P* < 0.05 for comparison of T2 compared with T3.

**TABLE 3:** Sperm quality parameters of study participants, in the lowest and highest tertiles of micronutrients status

Characteristics	Plasma folate (nmol/L)			RBCF (nmol/L)			Plasma vitamin B_12_ (pmol/L)			Plasma tHcy (μmol/L)		
	T1 (≤11.87)	T2 (>11.87‒<17.00)	T3 (≥17.00)	T1 (≤624.95)	T2 (>624.95‒<742.11)	T3 (≥742.11)	T1 (≤213.30)	T2 (>213.30‒<265.90)	T3 (≥265.90)	T1 (≤8.40)	T2 (>8.40‒<10.69)	T3 (≥10.69)
*n*	65	66	66	65	66	65	65	66	66	65	66	66
Sperm parameters												
Total sperm count (× 10^6^ spz.); median (P25-P75)	135.9 (64.2, 272.5)	170.1 (91.4, 265.0)	186.7 (117.8, 294.6)	150.0 (66.5, 310.4)	148.4 (101.5, 243.8)	203.9 (118.2, 312.5)	180.4 (90.9, 312.8)	180.9 (104.2, 281.8)	145.3 (89.4, 240.4)	180.5 (107.1, 319.6)	148.7 (105.1, 218.3)	170.1 (74.0, 272.0)
Sperm concentration (× 10^6^ spz./mL); median (P25-P75)	46.0 (25.1, 68.2)	47.6 (28.1, 84.1)	57.0 (33.1, 87.9)	44.3 (27.9, 69.0)	54.0 (28.1, 85.2)	52.6 (32.9, 91.6)	48.3 (31.2, 76.4)	58.4 (32.9, 85.9)	44.8 (26.6, 83.6)	47.3 (26.8, 85.6)	48.5 (32.1, 81.2)	51.2 (27.1, 75.7)
Sperm vitality (%); median (P25-P75)	80.5 (76.0, 87.5)	81.0 (74.5, 87.0)	83.8 (76.0, 91.4)	81.0 (73.5, 88.0)	83.0 (77.0, 90.5)	80.5 (72.5, 86.0)	80.8 (76.0, 86.4)	81.0 (73.6, 85.9)	84.5 (76.9, 91.0)	82.5 (75.0, 91.0)	81.5 (75.5, 86.0)	81.0 (76.1, 87.5)
Total motility (%); median (P25-P75)	59.4 (41.9, 71.8)	61.7 (47.7, 73.3)	61.5 (51.9, 73.6)	61.5 (45.2, 74.0)	61.2 (48.6, 71.0)	61.5 (50.0, 74.5)	66.5 (51.0, 74.0)	61.5 (48.6, 74.1)	57.2 (45.4, 70.2)	56.5 (46.4, 67.5)	66.8 (51.2, 74.4)^1^	61.8 (46.9, 72.5)
Progressive motility (%); median (P25-P75)	43.2 (26.6, 56.5)	41.3 (31.1, 57.3)	47.9 (37.5, 56.5)	45.0 (29.3, 57.5)	41.7 (30.7, 52.4)	50.5 (34.7, 57.5)^2^	48.0 (35.6, 56.6)	48.4 (32.8, 59.9)	41.3 (28.5, 51.3)	43.3 (30.8, 52.0)	52.0 (33.9, 60.4)	41.2 (28.8, 54.8)
Normal sperm morphology (%); median (P25-P75)	9.5 (5.0, 15.0)	8.0 (5.0, 14.0)	8.2 (5.0, 14.2)	7.5 (4.0, 14.0)	9.8 (6.1, 14.9)	11.2 (5.0, 16.6)	7.0 (5.0, 18.0)	10.0 (5.1, 14.4)	9.5 (5.0, 15.0)	8.0 (5.0, 13.0)	11.3 (5.1, 16.5)	7.5 (4.5, 15.0)
Total sperm count <39 × 10^6^ spz.; % (95% CI)	10.8 (5.3, 20.6)	10.6 (5.2, 20.3)	3.0 (0.8, 10.4)^1^^,^^2^	12.3 (6.4, 22.5)	7.6 (3.3, 16.5)	4.6 (1.6, 12.7)	6.2 (2.4, 14.8)	6.1 (2.4, 14.6)	12.1 (6.3, 22.1)	9.2 (4.3, 18.7)	6.1 (2.4, 14.6)	9.1 (4.2, 18.4)
Sperm concentration <15 × 10^6^ spz./mL; % (95% CI)	12.3 (6.4, 22.5)	13.6 (7.3, 23.9)	3.0 (0.8, 10.4)^1^	15.4 (8.6, 26.1)	6.1 (2.4, 14.6)	7.7 (3.3, 16.8)	7.7 (3.3, 16.8)	10.6 (5.2, 20.3)	10.6 (5.2, 20.3)	10.8 (5.3, 20.6)	7.6 (3.3, 16.5)	10.6 (5.2, 20.3)
Sperm vitality <58%; % (95% CI)	7.7 (3.3, 16.8)	3.1 (0.8, 10.5)	3.0 (0.8, 10.4)	7.7 (3.3, 16.8)	0.0 (0.0, 5.6)	6.2 (2.4, 14.8)	4.7 (1.6, 12.9)	4.5 (1.6, 12.5)	4.5 (1.6, 12.5)	4.6 (1.6, 12.7)	6.2 (2.4, 14.8)	3.0 (0.8, 10.4)
Total motility <40% motile; % (95% CI)	21.5 (13.3, 33.0)	6.1 (2.4, 14.6)	6.1 (2.4, 14.6)^1^	15.4 (8.6, 26.1)	9.1 (4.2, 18.4)	9.2 (4.3, 18.7)	6.2 (2.4, 14.8)	9.1 (4.2, 18.4)	18.2 (10.7, 29.1)	12.3 (6.4, 22.5)	6.1 (2.4, 14.6)	15.2 (8.4, 25.7)
Progressive motility <32% motile; % (95% CI)	35.4 (24.9, 47.5)	28.8 (19.3, 40.6)	16.7 (9.6, 27.4)^1^	33.8 (23.5, 46.0)	27.3 (18.0, 39.0)	20.0 (12.1, 31.3)	21.5 (13.3, 33.0)	21.2 (13.1, 32.5)	37.9 (27.1, 49.9)^1^	29.2 (19.6, 41.2)	21.2 (13.1, 32.5)	30.3 (20.6, 42.2)
Normal sperm morphology <4% normal; % (95% CI)	20.0 (12.1, 31.3)	9.2 (4.3, 18.7)	12.1 (6.3, 22.1)	21.5 (13.3, 33.0)	7.6 (3.3, 16.5)	12.5 (6.5, 22.8)	15.4 (8.6, 26.1)	15.2 (8.4, 25.7)	10.8 (5.3, 20.6)	10.8 (5.3, 20.6)	12.1 (6.3, 22.1)	18.5 (10.9, 29.6)
Altered seminogram^3^; % (95% CI)	52.3 (40.4, 64.0)	39.1 (28.1, 51.3)	28.8 (19.3, 40.6)^1^	50.8 (38.9, 62.5)	35.4 (24.9, 47.5)	34.4 (23.9, 46.6)	35.9 (25.3, 48.2)	33.3 (23.2, 45.3)	50.8 (38.9, 62.5)	44.6 (33.2, 56.7)	29.2 (19.6, 41.2)	46.2 (34.6, 58.1)

Continuous variables are reported as median (25th and 75th percentiles), and categorical variables as percentage (95% CI). *P* value was calculated by the Kruskal–Wallis test for continuous variables and then corrected for Bonferroni correction for multiple comparisons. χ^2^ test was used to compare categorical variables.

Abbreviations: CI, confidence interval; *n*, number of subjects; P25-P75, 25th and 75th percentiles; RBCF, red blood cell folate; T, tertile; tHcy, fasting total homocysteine; WHO, World Health Organization.

1 *P* < 0.05 for comparison of specific T compared with T1.

2 *P* < 0.05 for comparison of T2 compared with T3.

3 Participants who have 1 or more altered sperm quality parameters according to WHO 2010 thresholds.

### Genetic polymorphisms, B-vitamin status, and sperm quality

**FIGURE 1:**
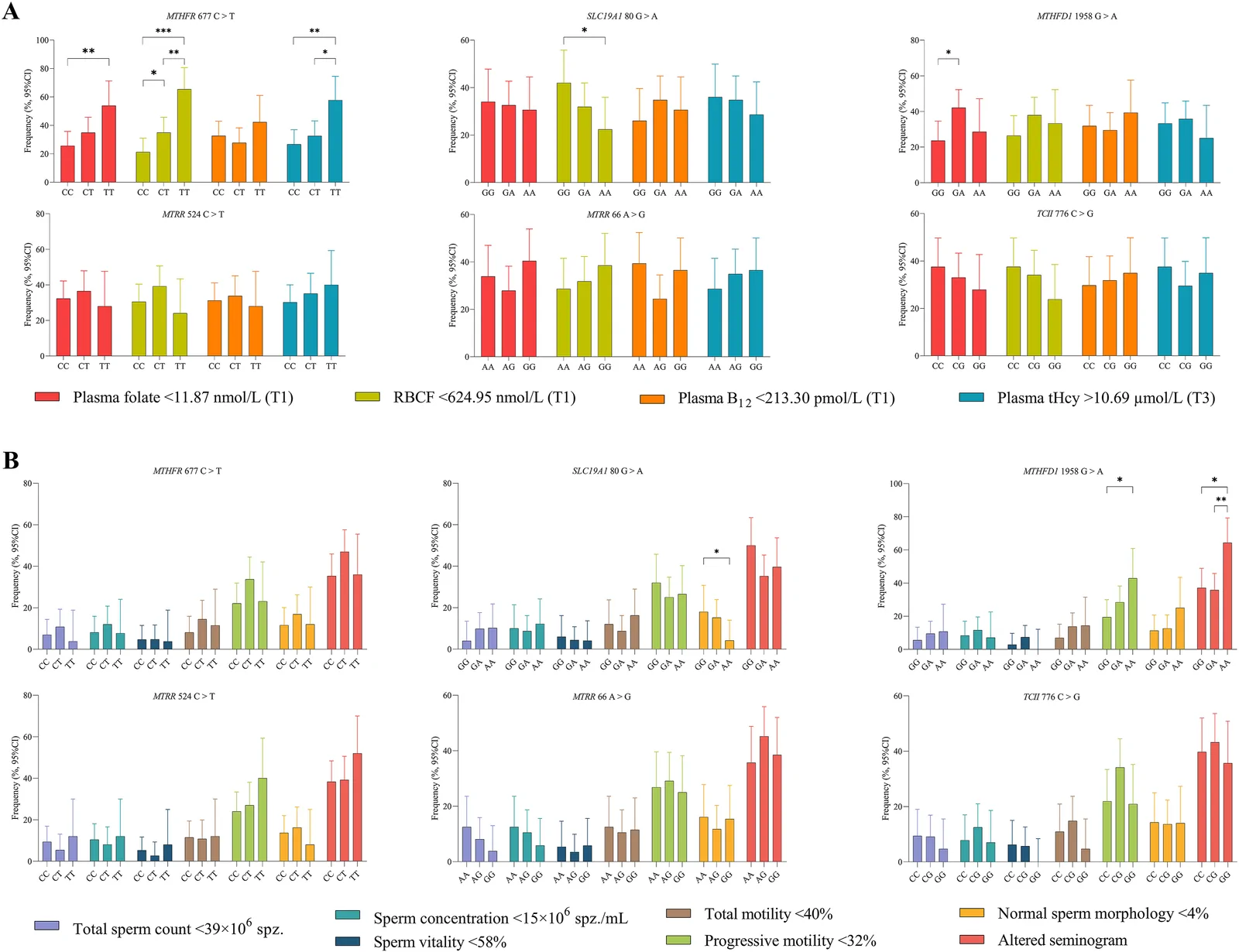
B-vitamin status and sperm quality parameters of the study participants according to genetic polymorphism. (A) Prevalence (percentage) and 95% CI of participants in the lowest plasma folate (<11.87 nmol/L), RBCF (<624.95 nmol/L), and plasma vitamin B_12_ (<213.30 pmol/L) tertiles, and in the highest plasma tHcy tertile (>10.69 μmol/L), stratified by genotype. (B) Prevalence (percentage) and 95% CI of participants with semen parameters below 2010 WHO reference limits, stratified by genotype. Differences between genotypes were assessed using a χ^2^ test. Altered seminogram was defined as the presence of 1 or more semen parameters below WHO 2010 reference limits. ^∗^*P* < 0.05, ^∗∗^*P* < 0.01, ^∗∗∗^*P* < 0.001. CI, confidence interval; *MTHFD1*, methylenetetrahydrofolate dehydrogenase-5,10-methylenetetrahydrofolate cyclohydrolase-10-formyltetrahydrofolate synthetase; MTHFR, methylenetetrahydrofolate reductase; *MTRR*, methionine transferase reductase; RBCF, red blood cell folate; *SLC19A1*, solute carrier family 19 (folate transporter) member 1; T, tertile; *TCII*, transcobalamin II; tHcy, fasting total homocysteine; WHO, World Health Organization.

Participants carrying the TT genotype of the *MTHFR* 677C>T polymorphism had lower folate status, a higher prevalence of folate deficiency, and higher tHcy compared to those with the CC genotype. No differences in sperm quality parameters were observed among the *MTHFR* 677C>T genotypes (Figure 1 and Supplemental Table 1).

Participants with the *SLC19A1* 80GG genotype had a higher prevalence of low RBCF (<625 nmol/L) and low normal sperm morphology (<4% normal forms) than those with the AA genotype (Figure 1 and Supplemental Table 2).

Participants with the *MTHFD1* 1958AA genotype had a higher prevalence of low progressive motility (<32% motile) and an altered seminogram compared to GG and GA carriers (Figure 1 and Supplemental Table 3).

No differences in status biomarkers and sperm quality were observed among the different *MTRR* 524C>T, *MTRR* 66A>G, and *TCII* 776C>G genotypes (Figure 1 and Supplemental Tables 4‒6).

### Correlation matrix between B-vitamin status and sperm quality parameters

The results of the Spearman correlation matrix between B-vitamin status biomarkers and sperm quality parameters are shown in Supplemental Figure 3. Plasma folate was positively correlated with total sperm count (ρ = 0.157, *P* < 0.05), RBCF with sperm concentration (ρ = 0.142, *P* < 0.05), and plasma vitamin B_12_ with sperm vitality (ρ = 0.141, *P* < 0.05). However, no correlation between plasma tHcy and sperm quality parameters was observed (all *P* > 0.05).

### Multivariable associations between B-vitamin status, genetic polymorphisms, and sperm quality

Associations between B-vitamin status biomarkers and sperm quality parameters are reported in Table 4. Compared with the highest tertile (reference), both the lowest tertile plasma folate and RBCF status were associated with lower total sperm count (B = ‒0.428, SE = 0.196, *R*^2^ = 0.136 and B = ‒0.459, SE = 0.183, *R*^2^ = 0.143, respectively). No associations were observed between plasma vitamin B_12_ or tHcy and any measured sperm quality parameters.

**TABLE 4:** Multiple linear regression analysis examining the associations between B-vitamins status biomarkers and the main sperm quality parameters.

Sperm quality parameters		Plasma folate status (nmol/L)^2^				RBCF status (nmol/L)^2^				Plasma vitamin B_12_ status (pmol/L)^2^				Plasma tHcy status (μmol/L)^2^			
		T1 (≤11.87) vs. T3 (>17.00)	T2 (>11.87‒<17.00) vs. T3 (>17.00)	*P-*trend	*R*^2^	T1 (≤624.95) vs. T3 (>742.11)	T2 (>624.95‒<742.11) vs. T3 (>742.11)	*P-*trend	*R*^2^	T1 (≤213.30) vs. T3 (>265.90)	T2 (>213.30‒<265.90) vs. T3 (>265.90)	*P-*trend	*R*^2^	T2 (>8.40‒<10.69) vs. T1 (<8.40)	T3 (≥10.69) vs. T1 (<8.40)	*P-*trend	*R*^2^
*n*		65	66			65	66			65	66			66	66		
Total sperm count (× 10^6^ spz.)^1^	Crude model	‒0.342 (0.172)^3^	‒0.286 (0.171)	0.104	0.023	‒0.346 (0.173)^3^	‒0.265 (0.172)	0.114	0.022	0.202 (0.173)	0.210 (0.172)	0.389	0.010	‒0.161 (0.173)	‒0.159 (0.173)	0.567	0.006
	Model 1	‒0.337 (0.180)	‒0.296 (0.172)	0.028	0.101	‒0.362 (0.171)^3^	‒0.312 (0.173)	0.021	0.106	0.247 (0.172)	0.238 (0.172)	0.020	0.106	‒0.036 (0.175)	‒0.120 (0.177)	0.079	0.078
	Model 2	‒0.428 (0.196)^3^	‒0.317 (0.179)	0.049	0.136	‒0.459 (0.183)^3^	‒0.323 (0.178)	0.035	0.143	0.267 (0.180)	0.218 (0.180)	0.129	0.116	‒0.040 (0.189)	‒0.149 (0.196)	0.417	0.116
Sperm concentration (× 10^6^ spz./mL)^1^	Crude model	‒0.307 (0.161)	‒0.276 (0.161)	0.113	0.022	‒0.305 (0.162)	‒0.120 (0.162)	0.170	0.018	0.086 (0.163)	0.160 (0.162)	0.615	0.005	‒0.014 (0.163)	‒0.036 (0.163)	0.975	0.001
	Model 1	‒0.351 (0.172)^3^	‒0.303 (0.164)	0.206	0.068	‒0.318 (0.164)	‒0.122 (0.166)	0.279	0.062	0.098 (0.165)	0.163 (0.165)	0.211	0.068	0.064 (0.167)	‒0.039 (0.170)	0.492	0.043
	Model 2	‒0.472 (0.188)^3^	‒0.365 (0.171)^3^	0.217	0.106	‒0.388 (0.177)^3^	‒0.118 (0.172)	0.346	0.094	0.126 (0.172)	0.142 (0.173)	0.488	0.081	0.041 (0.182)	‒0.077 (0.188)	0.869	0.076
Sperm vitality (%)^1^	Crude model	‒0.058 (0.028)^3^	‒0.032 (0.028)	0.127	0.021	0.012 (0.028)	0.060 (0.028)^3^	0.085	0.026	‒0.020 (0.029)	‒0.036 (0.028)	0.447	0.008	‒0.015 (0.029)	‒0.014 (0.028)	0.835	0.002
	Model 1	‒0.048 (0.030)	‒0.028 (0.029)	0.496	0.049	0.018 (0.029)	0.058 (0.029)^3^	0.369	0.057	‒0.018 (0.029)	‒0.045 (0.029)	0.480	0.050	‒0.024 (0.029)	‒0.010 (0.030)	0.664	0.035
	Model 2	‒0.045 (0.033)	‒0.025 (0.030)	0.546	0.079	0.019 (0.031)	0.058 (0.030)	0.418	0.089	‒0.008 (0.031)	‒0.035 (0.03)	0.63	0.072	‒0.018 (0.031)	‒0.002 (0.032)	0.583	0.103
Total motility (%)^1^	Crude model	‒0.108 (0.063)	‒0.028 (0.063)	0.213	0.016	‒0.096 (0.064)	‒0.025 (0.063)	0.299	0.012	0.094 (0.063)	0.042 (0.063)	0.333	0.011	0.079 (0.063)	‒0.007 (0.063)	0.314	0.012
	Model 1	‒0.129 (0.067)	‒0.046 (0.064)	0.145	0.075	‒0.096 (0.064)	‒0.021 (0.065)	0.206	0.068	0.096 (0.064)	0.030 (0.064)	0.059	0.09	0.086 (0.065)	0.006 (0.066)	0.181	0.064
	Model 2	‒0.150 (0.073)^3^	‒0.052 (0.066)	0.140	0.116	‒0.116 (0.068)	‒0.019 (0.066)	0.179	0.111	0.101 (0.066)	0.041 (0.066)	0.098	0.121	0.071 (0.068)	‒0.016 (0.071)	0.227	0.135
Progressive motility (%)	Crude model	‒5.929 (2.924)^3^	‒4.389 (2.912)	0.111	0.022	‒5.046 (2.945)	‒5.166 (2.933)	0.137	0.020	5.546 (2.917)	6.031 (2.906)^3^	0.073	0.027	6.188 (2.911)^3^	‒0.081 (2.911)	0.049	0.031
	Model 1	‒6.476 (3.143)^3^	‒5.107 (2.995)	0.322	0.059	‒5.142 (3.002)	‒4.937 (3.032)	0.413	0.053	6.327 (2.953)^3^	6.168 (2.943)^3^	0.034	0.098	6.640 (3.016)^3^	0.872 (3.062)	0.283	0.056
	Model 2	‒7.152 (3.419)3	‒5.396 (3.110)	0.353	0.093	‒5.133 (3.221)	‒4.631 (3.131)	0.468	0.084	6.338 (3.035)^3^	6.130 (3.040)^3^	0.065	0.129	5.601 (3.207)	0.049 (3.328)	0.453	0.113
Normal sperm forms (%)^1^	Crude model	0.029 (0.137)	0.084 (0.137)	0.824	0.002	‒0.150 (0.137)	0.085 (0.135)	0.223	0.016	‒0.018 (0.137)	0.032 (0.136)	0.934	0.001	0.126 (0.136)	0.012 (0.137)	0.597	0.005
	Model 1	0.112 (0.145)	0.100 (0.139)	0.388	0.056	‒0.147 (0.138)	0.087 (0.138)	0.259	0.065	0.060 (0.141)	0.067 (0.139)	0.404	0.055	0.110 (0.139)	0.054 (0.142)	0.312	0.055
	Model 2	0.177 (0.155)	0.127 (0.141)	0.183	0.112	‒0.074 (0.148)	0.155 (0.140)	0.14	0.119	0.079 (0.146)	0.035 (0.145)	0.568	0.077	0.120 (0.147)	0.096 (0.153)	0.361	0.123

Values are B**-**vitamin, nonstandardized coefficient, and SE. Crude model: unadjusted. Model 1 was adjusted for plasma folate (nanomoles per liter) (in the plasma vitamin B_12_ model), plasma vitamin B_12_ (picomoles per liter) (in the plasma folate and RBCF models), age (years), BMI, education (high school or less, college or higher education), smoking status (non-smoker, current smoker, and former smoker), physical activity (MET minutes per week), abstinence days (days), and plasma creatinine (micromoles per liter). For plasma tHcy, model 1 included all the above covariates except plasma folate (nanomoles per liter) and plasma vitamin B_12_ (picomoles per liter). Model 2 was further adjusted for relevant genetic polymorphisms, depending on the exposure variable. Specifically, plasma folate and RBCF model 2 were additionally adjusted for *MTHFR* 677CT compared with CC, *MTHFR* 677TT compared with CC, *SLC19A1* 80GA compared with GG, *SLC19A1* 80AA compared with GG, *MTHFD1* 1958GA compared with GG, and *MTHFD1* 1958AA compared with GG. Plasma vitamin B_12_ model 2 was additionally adjusted for *MTRR* 524CT compared with CC, *MTRR* 524TT compared with CC, *MTRR* 66AG compared with AA, *MTRR* 66GG compared with AA, *TCII* 776CG compared with CC, and *TCII* 776GG compared with CC. Plasma tHcy model 2 was additionally adjusted for all 6 SNPs.

Abbreviations: BMI, body mass index; MET, metabolic equivalent of task; *MTHFD1,* methylenetetrahydrofolate dehydrogenase-5,10-methylenetetrahydrofolate cyclohydrolase-10-formyltetrahydrofolate synthetase; *MTHFR,* methylenetetrahydrofolate reductase; *MTRR,* methionine transferase reductase; *n*, number of subjects; RBCF, red blood cell folate; SE, standard error; *SLC19A1,* solute carrier family 19 (folate transporter) member 1; SNP, single nucleotide polymorphism; T, tertile; *TCII,* transcobalamin II; tHcy, fasting total homocysteine.

1 Variables were natural log transformed to approximate a normal distribution.

2 T3 was used as the reference T for plasma folate, RBCF, and plasma vitamin B_12_, and T1 was used as the reference T for plasma tHcy.

3 *P* value <0.05 for comparison with reference T.

Associations between the SNPs and sperm quality parameters are reported in Supplemental Tables 7‒12. Participants with the *MTHFD1* 1958AA compared to GG genotype had reduced progressive motility (B = ‒7.732%, SE = 3.782, *R*^2^ = 0.100) (Supplemental Table 9). No associations were observed between *MTHFR* 677C>T, *SLC19A1* 80G>A, *MTRR* 524C>T, *MTRR* 66A>G, and *TCII* 776C>G polymorphisms and sperm quality parameters. The previously observed association between *SLC19A1* 80G>A and sperm morphology was not retained in multivariate analysis.

**FIGURE 2:**
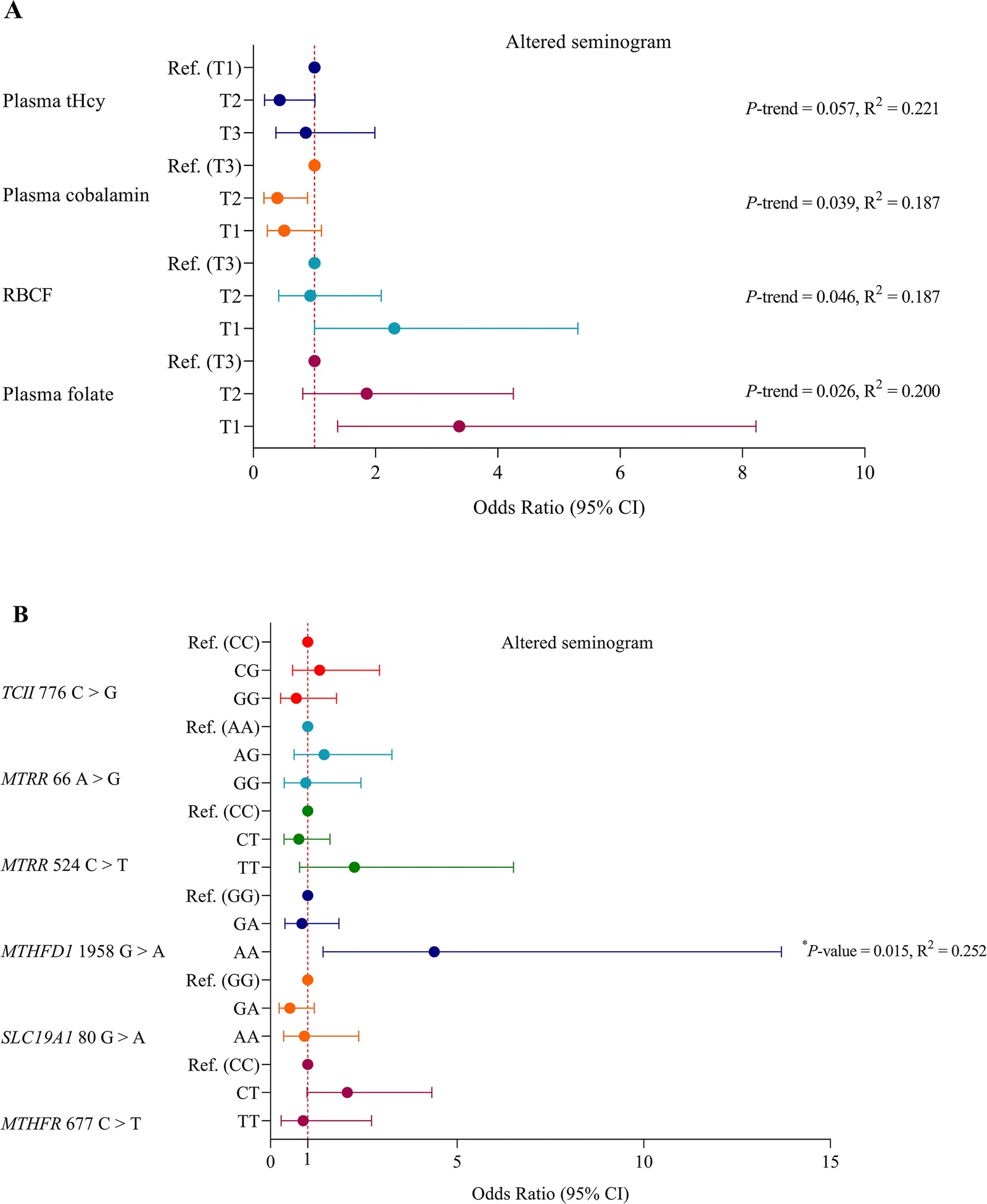
Associations between B-vitamin status, SNPs, and odds for an altered seminogram. (A) Odds ratios and 95% CI for altered seminogram according to tertiles of plasma folate, RBCF, plasma vitamin B_12_, and plasma tHcy. (B) Odds ratios and 95% CI for altered seminogram according to genotypes. All odds ratios reported are from fully adjusted binary logistic regression models, including all covariates specified in the Methods section. Altered seminogram was defined as the presence of 1 or more semen parameters below WHO 2010 reference limits. ^∗^*P* value refers to binary logistic model. CI, confidence interval; *MTHFD1*, methylenetetrahydrofolate dehydrogenase-5,10-methylenetetrahydrofolate cyclohydrolase-10-formyltetrahydrofolate synthetase; MTHFR, methylenetetrahydrofolate reductase; *MTRR*, methionine transferase reductase; RBCF, red blood cell folate; Ref., reference; *SLC19A1*, solute carrier family 19 (folate transporter) member 1; SNP, single nucleotide polymorphism; T, tertile; *TCII*, transcobalamin II; tHcy, fasting total homocysteine; WHO, World Health Organization.

The odds [OR (95% CI)] of altered seminogram according to B-vitamin status and polymorphism are shown in Figure 2. Lowest compared with highest plasma folate and RBCF tertile status were each associated with a higher probability of an altered seminogram (OR: 3.4; 95% CI: 1.4, 8.2 and OR: 2.3; 95% CI: 1.0, 5.3, respectively). Conversely, lowest compared with highest tertile plasma vitamin B_12_ and highest compared with lowest tertile plasma tHcy status were not associated with altered seminogram.

The *MTHFD1* 1958AA genotype, compared with the GG genotype, was associated with an increased probability of an altered seminogram (OR: 4.4; 95% CI: 1.4, 13.7). However, no associations with the seminogram were observed for the other polymorphisms (Figure 2).

## Discussion

Lowest compared with highest tertile RBCF status was associated with reduced sperm count in healthy young adult males living in a region free of mandatory folic acid food fortification. Multivariate regression analyses confirmed that being in the lowest compared with highest status groups for plasma folate and RBCF status were associated with increased probability of an altered seminogram. No associations were observed between plasma vitamin B_12_ or plasma tHcy status and sperm quality. The *MTHFD1* 1958AA compared with GG genotype was associated with an 8% reduction in progressive sperm motility and an increased probability of an altered seminogram.

Evidence from randomized controlled trials investigating the effect of folic acid supplementation on sperm quality is inconsistent. A European double-blind, placebo-controlled trial in subfertile males of 5 mg/d folic acid and 66 mg/d zinc sulfate, alone or in combination, resulted in an increased total normal sperm count in the combined arm of the trial. A similar trend was observed in fertile males [20]. Conversely, a randomized clinical trial in the United States couples undergoing infertility treatment reported no beneficial effects of combined folic acid (5 mg/d) and zinc (30 mg/d) supplementation on semen quality [37]. Baseline differences in the underlying prevalence and causes of male infertility as well as folate status may explain the different results of these studies.

The present study showed that participants in the lowest folate status categories, compared to the highest, had lower sperm count. Future research should investigate whether low folate status is causally associated with low sperm count. If this were the case, we hypothesize that impaired nucleotide synthesis, DNA repair, and epigenetic regulation [1,2] or uracil misincorporation during DNA synthesis [38] and DNA damage [39] could be explored as potential mechanisms affecting sperm development and genomic integrity.

Plasma folate and RBCF concentrations are strongly correlated but provide complementary information on different aspects of folate status. Plasma folate primarily reflects recent dietary intake, and a single measurement may not reliably distinguish between low recent folate consumption and prolonged deficiency. On the contrary, RBCF concentrations reflect long-term folate intake [40,41] and more reliably indicate the longstanding folate reserves supporting spermatogenesis, a process that spans ∼70 ± 4 d in humans [18]. We observed consistent associations between both indicators of folate status and sperm count and altered seminogram, reinforcing our hypothesis that folate status is associated with spermatogenesis and a healthy seminogram.

No associations were observed between plasma vitamin B_12_ status and any sperm quality parameters in our study population. This contrasts with prior studies based on different populations and contexts. A prospective Dutch study of 73 males participating in an in vitro fertilization (IVF)/ intracytoplasmic sperm injection (ICSI) program reported higher plasma vitamin B_12_ status [median (range), 317 (141‒696) pmol/L] than our study, and seminal plasma vitamin B_12_ concentrations were positively correlated with sperm concentration [42]. In Spain, 20 infertile males with known asthenoteratozoospermia received a daily multivitamin formulation containing 1 μg of cobalamin in combination with L-carnitine, vitamins C and E, coenzyme Q10, folate, zinc, and selenium for 3 mo. Improvements were reported in sperm quality parameters after supplementation [43]. An Indian cross-sectional study of 52 infertile males reported that low serum vitamin B_12_ status (<200 pg/mL or 148 pmol/L) was associated with reduced sperm count and abnormal sperm morphology [44]. Conversely, our participants were generally healthy males not selected for infertility, varicocele, or abnormal sperm parameters, and their plasma vitamin B_12_ status was mostly normal. Cobalamin, a cofactor for methionine synthase in the homocysteine to methionine remethylation pathway, is crucial for S-adenosylmethionine (SAM) production and DNA methylation [31]. SAM is a methyl donor for DNA methylation and numerous transmethylation reactions involved in spermatogenesis, sperm maturation, and epigenetic programming [44]. The absence of associations between cobalamin status and sperm quality in our study may reflect the adequate cobalamin concentrations of the population.

The lower probability of altered seminogram observed in participants in the mid tertile of plasma vitamin B_12_, compared with the highest, may partly reflect underlying dietary and lifestyle differences across plasma vitamin B_12_ tertiles. Individuals in the highest tertile consumed more meat and less fruit and vegetables compared to the mid and lowest tertiles. Thus, dietary and lifestyle characteristics of this category may be confounding factors. This nuance highlights the inherent and recognized limitations of using a single vitamin B_12_ biomarker to ascertain cobalamin status.

We observed no difference in sperm quality parameters between participants with tHcy ≥10.7 μmol/L compared to the reference group (tHcy ≤8.4 μmol/L). Previously, we reported that first-trimester tHcy ≥P90 (7.1 μmol/L) is associated with double the probability of unexplained early pregnancy miscarriage, but this association is unlikely to be independent of folate status and the *MTHFR* 677TT genotype [3]. Similarly, in females undergoing IVF/ICSI, tHcy ≥9.30 μmol/L was associated with lower implantation and clinical pregnancy rates, particularly among carriers of the *MTHFR* 677TT genotype [45]. Few studies have investigated the association of elevated tHcy with infertility parameters in males. Albeit, investigating different outcomes to our study, in a retrospective cohort study on IVF/ICSI, male tHcy was not associated with embryo quality, clinical pregnancy, or pregnancy outcomes [46]. On the contrary, an observational study of males from couples undergoing IVF reported that seminal plasma tHcy was positively associated with sperm count, but tHcy concentrations were not reported [19].

The *MTHFD1* 1958AA genotype, affecting 14.4% of the participants, was associated with reduced sperm motility and overall altered semen parameters. This confirms the results of previous case-control studies of infertile males and controls. An Algerian study reported a higher risk of infertility in both variant genotypes compared with the GG genotype [47], and an Iranian study reported a higher frequency of the AA genotype among cases compared with controls [48]. Conversely, a case-control study in a Caucasian Russian population reported no association between the *MTHFD1* 1958G>A polymorphism and idiopathic male infertility [49]. A preliminary association with azoospermia was attenuated after correction for multiple comparisons. MTHFD1 generates 10-formyl-tetrahydrofolate, an essential one-carbon donor required for purine biosynthesis [1,2]. Although the arginine to glutamine substitution does not alter basal enzyme kinetics [50], it reduces enzyme stability and results in a ∼25% decrease in de novo purine synthesis [50]. Given the high nucleotide demand of spermatogenesis, we hypothesize that even a moderate reduction in de novo purine availability may compromise germ cell proliferation and DNA synthesis, thereby negatively affecting sperm production and quality.

Approximately 70% of folate-sensitive neural-tube defects are preventable through periconceptional folic acid supplementation in females, highlighting the essential role of folate-dependent one-carbon metabolism in early embryonic development [51,52]. To date, this preventive effect has been almost exclusively attributed to maternal folate status, whereas the potential contribution of paternal folate status has received limited attention. Experimental evidence indicates that folate deficiency in the father is associated with adverse offspring outcomes, accompanied by alterations in the father’s sperm DNA integrity and epigenetic programming of the offspring [53]. Previous studies have reported associations between sperm DNA damage and pregnancy loss and embryo malformations, supporting the biological plausibility that compromised paternal folate status may influence early developmental processes [54,55]. However, whether impaired sperm quality or altered folate-dependent mechanisms in the father contribute to folate-sensitive pregnancy complications, such as neural-tube defects, remains to be explored.

Strengths of our study include a thorough assessment of folate and vitamin B_12_ status as well as associated SNPs and their association with male fertility parameters in a study of males free of diagnosed infertility and in the absence of environmental exposures known to enhance folate status, such as mandatory folic acid supplementation or widespread B-vitamin use. Biological samples were collected and processed according to strict handling, processing, and storage protocols, specifically for determinations of the study. All measurements were carried out by gold standard methods. Both plasma and RBCF status were assessed. Semen analysis was performed using the standardized computer-assisted sperm analysis SCA system methods. The study also had limitations. Approximately 65% of the participants were highly educated. However, we considered this potential confounding variable in our analysis. Although several statistically significant associations were observed, the effect sizes were modest, and the explanatory power of the multivariable models was limited. Some CIs were relatively wide, indicating uncertainty in the estimates. Our multivariate model included the core established predictors of male fertility but only explained ≤15% of the variability in sperm count and motility. Few studies to date have reported the variability or even the significance of the models providing much of the evidence in the literature to date.

Although our results show that folate status is associated with sperm count and quality in healthy young males, they also suggest that other contributing factors to male fertility remain unknown or unmeasured. In particular, we did not measure hormonal profiles or reactive oxygen species. Clinically relevant endocrine factors have been reported in 2‒10% of male infertility cases [56]. Nevertheless, in our study of males without previously diagnosed infertility, we do not expect altered endocrine factors to be an important confounder in our analysis.

In conclusion, in young males with no history of infertility, having plasma folate status or RBCF status in the lowest compared to the highest tertile was associated with reduced sperm count. Plasma vitamin B_12_ and tHcy status were not associated with sperm quality parameters. Additionally, the *MTHFD1* 1958AA compared to the GG genotype was associated with lower sperm motility and a higher probability of an altered seminogram.

## Author contributions

The authors’ responsibilities were as follows – AE-A, AS-H, MM: designed research; AE-A, AS-H, CR-R: analyzed data and performed statistical analysis; AE-A: wrote the manuscript; AS-H, CR-R, LS-C, RA, JS-S, NB, AM, PU, MM: reviewed and edited the manuscript; and all authors read and approved the final manuscript.

## Data availability

The data underlying this article is available from the corresponding author upon request.

## Declaration of generative AI and AI-assisted technologies in the writing process

The author(s) declare that no generative AI or AI-assisted technologies were used in the writing of this manuscript.

## Funding

This research received financial support from several sources: the Spanish government’s official agency for biomedical research, Instituto de Salud Carlos III (ISCIII), through the Fondo de Investigación para la Salud (FIS) co-founded by the European Union ERDF/ESF under the initiatives “A way to make Europe” and “Investing in your future” (PI21/01447, PI25/00603); and the Diputació de Tarragona (2021/11-No.Exp. 8004330008-2021-0022642). AE-A is a predoctoral researcher supported by a grant from Universitat Rovira i Virgili and Diputació de Tarragona (2022PMF-PIPF-06). JS-S is a distinguished senior researcher supported by the ICREA Academia Program. The funding sources had no role in the design of the study, in the collection, analysis, or interpretation of the data, in the writing of the manuscript, or in the decision to submit the manuscript for publication.

## Conflict of interest

The authors declare no conflicts of interest.
