# Supplementary material for: Low folate status and related polymorphisms are associated with lower sperm quality in healthy males: The Lifestyle and Environmental Determinants of Seminogram and Other Male Fertility-Related Parameters (Led-Fertyl) cross-sectional study
Source: Am J Clin Nutr. 2026 Jun 6;124(2):101385. doi: 10.1016/j.ajcnut.2026.101385 (PMC13494078; doi:10.1016/j.ajcnut.2026.101385)

Low folate status and related polymorphisms are associated with lower sperm quality in healthy men: The Led-Fertyl cross-sectional study. Eigbefoh-Addeh et al.

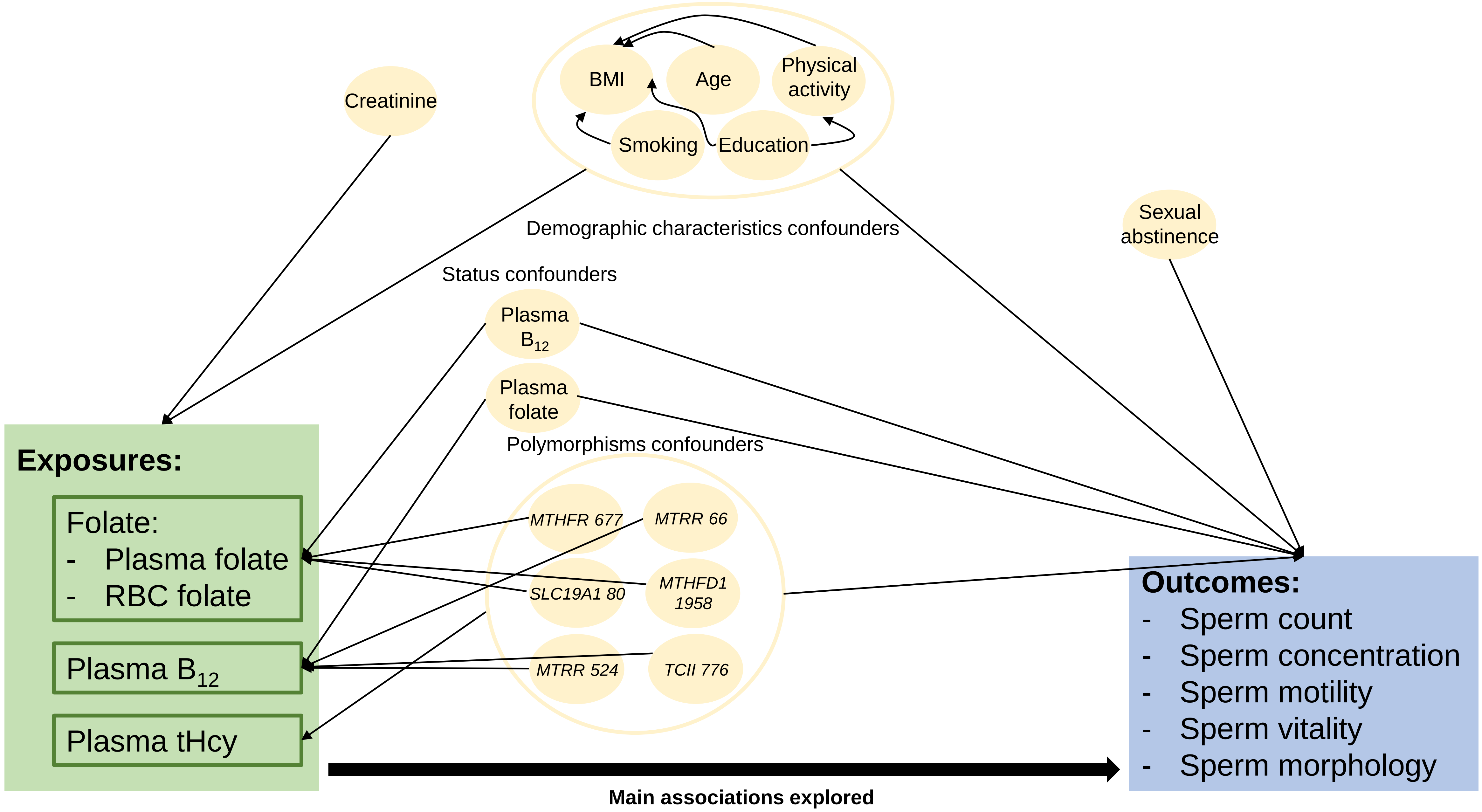

Supplement: Supplementary file 1 [file mmc2.pdf]
