# Supplementary material for: Low folate status and related polymorphisms are associated with lower sperm quality in healthy males: The Lifestyle and Environmental Determinants of Seminogram and Other Male Fertility-Related Parameters (Led-Fertyl) cross-sectional study
Source: Am J Clin Nutr. 2026 Jun 6;124(2):101385. doi: 10.1016/j.ajcnut.2026.101385 (PMC13494078; doi:10.1016/j.ajcnut.2026.101385)
Supplement: Supplementary file 4 [file mmc1.docx]

Low folate status and related polymorphisms are associated with lower sperm quality in healthy men: The Led-Fertyl cross-sectional study. Eigbefoh-Addeh et al.

**SUPPLEMENTARY TABLES**

**Supplementary Table S1.** Baseline characteristics of the study population according to *MTHFR* 677 C>T polymorphism.

**Supplementary Table S2.** Baseline characteristics of the study population according to *SLC19A1* 80 G>A polymorphism.

**Supplementary Table S3.** Baseline characteristics of the study population according to *MTHFD1* 1958 G>A polymorphism.

**Supplementary Table S4.** Baseline characteristics of the study population according to *MTRR* 524 C>T polymorphism.

**Supplementary Table S5.** Baseline characteristics of the study population according to *MTRR* 66 A>G polymorphism.

**Supplementary Table S6.** Baseline characteristics of the study population according to *TCII* 776 C>G polymorphism.

**Supplementary Table S7.** Multiple linear regression analysis to test the associations between *MTHFR* 677 C>T polymorphism and sperm quality parameters.

**Supplementary Table S8.** Multiple linear regression analysis to test the associations between *SLC19A1* 80 G>A polymorphism and sperm quality parameters.

**Supplementary Table S9.** Multiple linear regression analysis to test the associations between *MTHFD1* 1958 G>A polymorphism and sperm quality parameters.

**Supplementary Table S10.** Multiple linear regression analysis to test the associations between *MTRR* 524 C>T polymorphism and sperm quality parameters.

**Supplementary Table S11.** Multiple linear regression analysis to test the associations between *MTRR* 66 A>G polymorphism and sperm quality parameters.

**Supplementary Table S12.** Multiple linear regression analysis to test the associations between *TCII* 776 C>G polymorphism and sperm quality parameters.

**SUPPLEMENTARY FIGURE CAPTIONS**

**Supplementary Figure 1.** The directed acyclic graph (DGA) for our assumptions of the analysis of associations between folate, cobalamin, and homocysteine status and single nucleotide polymorphisms (SNPs) influencing folate availability and its role in DNA synthesis with sperm quality.

Exposures in green, confounders in yellow, outcomes in blue.

**Supplementary Figure 2.** Flowchart of included participants.

**Supplementary Figure 3.** Spearman correlation matrix (rho values) between B-vitamins status biomarkers and sperm quality parameters in 197 participants.

Post-hoc Bonferroni correction for multiple comparisons of *P*-values was applied. ^*^*P* <0.05, ^**^*P* <0.01, ^***^*P* <0.001.

Abbreviations. B_12_, cobalamin; RBCF, red blood cell folate; tHcy, fasting total homocysteine.

**Supplementary Table S1.** Baseline characteristics of the study population according to *MTHFR* 677 C>T polymorphism.

|  | ***MTHFR* 677 C>T polymorphism** | | | | |
| --- | --- | --- | --- | --- | --- |
|  | **N** | **CC** | **CT** | **TT** | ***P*-value** |
|  |  | **n = 86** | **n = 83** | **n = 26** |  |
| **Demographic parameters** |  |  |  |  |  |
| Age (years); median (P25-P75) | 195 | 28.0 (24.0, 31.0) | 29.0 (23.0, 33.0) | 30.0 (26.0, 32.0) | 0.239 |
| BMI (kg/m^2^); median (P25-P75) | 195 | 24.1 (22.1, 25.5) | 24.5 (22.3, 26.6) | 24.5 (22.2, 26.0) | 0.715 |
| Physical activity (MET min/week); median (P25-P75) | 195 | 3302 (1678, 5121) | 3730 (2061, 5740) | 3546 (1791, 5146) | 0.866 |
| Sexual abstinence (days); median (P25-P75) | 195 | 4.0 (3.0, 4.4) | 4.0 (3.0, 5.0) | 4.0 (3.0, 5.0) | 0.580 |
| Smoking status; % (95% CI) | 183 |  | | | |
| Non-smoker | 134 | 72.2 (61.4, 80.8) | 77.2 (66.8, 85.1) | 64.0 (44.5, 79.8) | 0.684 |
| Current smoker | 24 | 15.2 (8.9, 24.7) | 10.1 (5.2, 18.7) | 16.0 (6.4, 34.7) |  |
| Former smoker | 25 | 12.7 (7.0, 21.8) | 12.7 (7.0, 21.8) | 20.0 (8.9, 39.1) |  |
| Education; % (95% CI) | 195 |  | | | |
| High school or less | 70 | 40.7 (30.9, 51.3) | 32.5 (23.4, 43.2) | 30.8 (16.5, 50.0) | 0.457 |
| College or high education | 125 | 59.3 (48.7, 69.1) | 67.5 (56.8, 76.6) | 69.2 (50.0, 83.5) |  |
| **Blood parameters** |  |  |  |  |  |
| Creatinine (µmol/L); median (P25-P75) | 194 | 82.2 (75.2, 89.1) | 80.5 (75.2, 88.4) | 85.3 (75.4, 91.1) | 0.798 |
| Plasma folate (nmol/L); median (P25-P75) | 195 | 15.3 (11.7, 18.3) | 14.2 (11.0, 17.7) | 11.0 (7.7, 13.7)^a^ | <0.001 |
| RBCF (nmol/L); median (P25-P75) | 194 | 701.7 (647.1, 833.5) | 677.3 (562.3, 759.2) | 572.0 (433.5, 705.4)^a^ | 0.051 |
| Plasma B_1_**_2_** (pmol/L); median (P25-P75) | 195 | 245.6 (207.5, 281.3) | 236.1 (208.9, 291.7) | 237.4 (174.7, 280.7) | 0.787 |
| Plasma tHcy (µmol/L); median (P25-P75) | 195 | 9.0 (7.5, 10.7) | 9.8 (7.8, 11.4) | 11.7 (9.8, 14.7)^a^ | 0.001 |
| Plasma folate <7 nmol/L; % (95% CI) | 195 | 2.3 (0.6, 8.1) | 2.4 (0.7, 8.4) | 19.2 (8.5, 37.9)^a, b^ | <0.001 |
| RBCF <340 nmol/L; % (95% CI) | 194 | 2.4 (0.6, 8.2) | 1.2 (0.2, 6.5) | 7.7 (2.1, 24.1) | 0.187 |
| Plasma B_1_**_2_** <148 pmol/L; % (95% CI) | 195 | 1.2 (0.2, 6.3) | 1.2 (0.2, 6.5) | 3.8 (0.7, 18.9) | 0.590 |
| Plasma tHcy >15 µmol/L; % (95% CI) | 195 | 2.3 (0.6, 8.1) | 3.6 (1.2, 10.1) | 23.1 (11.0, 42.1)^a, b^ | <0.001 |
| **Sperm parameters** |  |  |  |  |  |
| Total sperm count (x10^6^ spz.); median (P25-P75) | 195 | 154.9 (103.7, 291.9) | 168.4 (83.5, 269.6) | 172.3 (94.1, 270.7) | 0.902 |
| Sperm concentration (x10^6^ spz./mL); median (P25-P75) | 195 | 48.5 (28.7, 81.8) | 48.6 (29.3, 80.5) | 51.6 (30.1, 91.9) | 0.902 |
| Sperm vitality (%); median (P25-P75) | 194 | 82.5 (76.0, 88.5) | 81.5 (75.5, 87.5) | 81.0 (76.2, 88.9) | 0.874 |
| Total motility (%); median (P25-P75) | 195 | 60.9 (49.2, 72.1) | 61.0 (46.2, 74.2) | 70.9 (51.9, 77.5) | 0.215 |
| Progressive motility (%); median (P25-P75) | 195 | 47.0 (33.9, 55.9) | 44.5 (26.1, 57.0) | 51.7 (32.9, 59.4) | 0.424 |
| Normal sperm morphology (%); median (P25-P75) | 194 | 10.5 (5.6, 15.4) | 7.5 (4.5, 14.0) | 9.5 (5.0, 20.5) | 0.280 |
| Total sperm count <39×10^6^ spz.; % (95% CI) | 195 | 7.0 (3.2, 14.4) | 10.8 (5.8, 19.3) | 3.8 (0.7, 18.9) | 0.450 |
| Sperm concentration <15×10^6^ spz./mL; % (95% CI) | 195 | 8.1 (4.0, 15.9) | 12.0 (6.7, 20.8) | 7.7 (2.1, 24.1) | 0.709 |
| Sperm vitality <58%; % (95% CI) | 194 | 4.7 (1.8, 11.5) | 4.8 (1.9, 11.7) | 3.8 (0.7, 18.9) | 1.000 |
| Total motility <40% motile; % (95% CI) | 195 | 8.1 (4.0, 15.9) | 14.5 (8.5, 23.6) | 11.5 (4.0, 29.0) | 0.446 |
| Progressive motility <32% motile; % (95% CI) | 195 | 22.1 (14.6, 31.9) | 33.7 (24.5, 44.4) | 23.1 (11.0, 42.1) | 0.207 |
| Normal sperm morphology <4% normal; % (95% CI) | 194 | 11.6 (6.4, 20.1) | 16.9 (10.3, 26.3) | 12.0 (4.2, 30.0) | 0.625 |
| Altered seminogram^c^; % (95% CI) | 193 | 35.3 (26.0, 45.9) | 47.0 (36.6, 57.6) | 36.0 (20.2, 55.5) | 0.270 |
| **Polymorphism** |  |  | | | |
| *SLC19A1* 80 G>A; % (95% CI) | 191 |  | | | |
| GG | 50 | 27.1 (18.8, 37.3) | 24.7 (16.6, 35.1) | 28.0 (14.3, 47.6) | 0.450 |
| GA | 92 | 42.4 (32.4, 53.0) | 55.6 (44.7, 65.9) | 44.0 (26.7, 62.9) |  |
| AA | 49 | 30.6 (21.8, 41.0) | 19.8 (12.5, 29.7) | 28.0 (14.3, 47.6) |  |
| *MTHFD1* 1958 G>A; % (95% CI) | 195 |  | | | |
| GG | 72 | 38.4 (28.8, 48.9) | 37.3 (27.7, 48.1) | 30.8 (16.5, 50.0) | 0.864 |
| GA | 95 | 45.3 (35.3, 55.8) | 50.6 (40.1, 61.1) | 53.8 (35.5, 71.2) |  |
| AA | 28 | 16.3 (10.0, 25.5) | 12.0 (6.7, 20.8) | 15.4 (6.1, 33.5) |  |
| *MTRR* 524 C>T; % (95% CI) | 195 |  | | | |
| CC | 96 | 50.0 (39.7, 60.3) | 47.0 (36.6, 57.6) | 53.8 (35.5, 71.2) | 0.960 |
| CT | 74 | 36.0 (26.7, 46.6) | 41.0 (31.0, 51.7) | 34.6 (19.4, 53.8) |  |
| TT | 25 | 14.0 (8.2, 22.8) | 12.0 (6.7, 20.8) | 11.5 (4.0, 29.0) |  |
| *MTRR* 66 A>G; % (95% CI) | 194 |  | | | |
| AA | 56 | 33.7 (24.6, 44.2) | 24.4 (16.4, 34.7) | 26.9 (13.7, 46.1) | 0.501 |
| AG | 86 | 45.3 (35.3, 55.8) | 43.9 (33.7, 54.7) | 42.3 (25.5, 61.1) |  |
| GG | 52 | 20.9 (13.7, 30.7) | 31.7 (22.6, 42.4) | 30.8 (16.5, 50.0) |  |
| *TCII* 776 C>G; % (95% CI) | 195 |  | | | |
| CC | 64 | 33.7 (24.6, 44.2) | 31.3 (22.4, 41.9) | 34.6 (19.4, 53.8) | 0.884 |
| CG | 88 | 41.9 (32.0, 52.4) | 47.0 (36.6, 57.6) | 50.0 (32.1, 67.9) |  |
| GG | 43 | 24.4 (16.6, 34.5) | 21.7 (14.2, 31.7) | 15.4 (6.1, 33.5) |  |

Continuous variables are reported as median (25^th^, 75^th^ percentile) and categorical variables as percentage (95% confidence interval). *P*-value was calculated by Kruskal-Wallis test for continuous variables and then corrected for Bonferroni correction for multiple comparisons. Chi-square test was used to compare categorical variables. ^a^*P*<0.05 for comparison of specific genotype versus normal homozygote. ^b^*P*<0.05 for comparison of heterozygote versus variant homozygote. ^c^Participants who have one or more altered sperm quality parameters according to WHO 2010 thresholds.

Abbreviations: %, percentage; BMI, body mass index; MET, metabolic equivalent of task; min, minute; MTHFD1, methylenetetrahydrofolate dehydrogenase-5,10-methylenetetrahydrofolate cyclohydrolase-10-formyltetrahydrofolate synthetase; MTHFR, methylenetetrahydrofolate reductase; MTRR, methionine transferase reductase; n, number of subjects; RBCF, red blood cell folate; SLC19A1, solute carrier family 19 (folate transporter) member 1; TCII, transcobalamin II; tHcy, fasting total homocysteine.

**Supplementary Table S2.** Baseline characteristics of the study population according to *SLC19A1* 80 G>A polymorphism.

|  | ***SLC19A1* 80 G>A polymorphism** | | | | |
| --- | --- | --- | --- | --- | --- |
|  | **N** | **GG** | **GA** | **AA** | ***P*-value** |
|  |  | **n = 50** | **n = 92** | **n = 49** |  |
| **Demographic parameters** |  |  |  |  |  |
| Age (years); median (P25-P75) | 191 | 29.0 (24.0, 34.5) | 28.5 (25.0, 32.2) | 28.0 (22.0, 31.0) | 0.984 |
| BMI (kg/m^2^); median (P25-P75) | 191 | 24.6 (22.7, 26.3) | 24.4 (21.8, 26.0) | 23.8 (21.7, 24.9) | 0.396 |
| Physical activity (MET min/week); median (P25-P75) | 191 | 2890 (1525, 4770) | 3716 (1923, 5319) | 3656 (1965, 5521) | 0.279 |
| Sexual abstinence (days); median (P25-P75) | 191 | 4.0 (3.0, 5.0) | 4.0 (3.0, 4.5) | 4.0 (3.0, 5.0) | 0.529 |
| Smoking status; % (95% CI) | 179 |  | | | |
| Non-smoker | 131 | 77.1 (63.5, 86.7) | 67.5 (56.8, 76.6) | 79.2 (65.7, 88.3) | 0.615 |
| Current smoker | 23 | 10.4 (4.5, 22.2) | 15.7 (9.4, 25.0) | 10.4 (4.5, 22.2) |  |
| Former smoker | 25 | 12.5 (5.9, 24.7) | 16.9 (10.3, 26.3) | 10.4 (4.5, 22.2) |  |
| Education; % (95% CI) | 191 |  | | | |
| High school or less | 66 | 26.0 (15.9, 39.6) | 33.7 (24.9, 43.8) | 44.9 (31.9, 58.7) | 0.138 |
| College or high education | 125 | 74.0 (60.4, 84.1) | 66.3 (56.2, 75.1) | 55.1 (41.3, 68.1) |  |
| **Blood parameters** |  |  |  |  |  |
| Creatinine (µmol/L); median (P25-P75) | 190 | 82.7 (73.6, 89.1) | 82.2 (76.9, 89.7) | 81.3 (74.3, 87.5) | 0.873 |
| Plasma folate (nmol/L); median (P25-P75) | 191 | 14.2 (11.2, 17.6) | 14.1 (10.3, 18.0) | 14.0 (11.1, 18.1) | 0.879 |
| RBCF (nmol/L); median (P25-P75) | 190 | 659.0 (544.8, 760.1) | 679.3 (588.4, 753.6) | 700.6 (647.1, 888.4) | 0.539 |
| Plasma B_1_**_2_** (pmol/L); median (P25-P75) | 191 | 240.4 (211.4, 287.7) | 232.1 (205.4, 278.9) | 254.3 (198.2, 294.0) | 0.640 |
| Plasma tHcy (µmol/L); median (P25-P75) | 191 | 9.5 (7.6, 11.6) | 9.8 (8.1, 11.6) | 9.5 (7.8, 10.8) | 0.933 |
| Plasma folate <7 nmol/L; % (95% CI) | 191 | 6.0 (2.1, 16.2) | 4.3 (1.7, 10.7) | 2.0 (0.4, 10.7) | 0.588 |
| RBCF <340 nmol/L; % (95% CI) | 190 | 4.0 (1.1, 13.5) | 2.2 (0.6, 7.7) | 0.0 (0.0, 7.3) | 0.375 |
| Plasma B_1_**_2_** <148 pmol/L; % (95% CI) | 191 | 0.0 (0.0, 7.1) | 3.3 (1.1, 9.2) | 0.0 (0.0, 7.3) | 0.440 |
| Plasma tHcy >15 µmol/L; % (95% CI) | 191 | 6.0 (2.1, 16.2) | 6.5 (3.0, 13.5) | 4.1 (1.1, 13.7) | 0.923 |
| **Sperm parameters** |  |  |  |  |  |
| Total sperm count (x10^6^ spz.); median (P25-P75) | 191 | 186.7 (112.4, 283.0) | 154.2 (84.5, 287.8) | 145.6 (101.5, 272.5) | 0.398 |
| Sperm concentration (x10^6^ spz./mL); median (P25-P75) | 191 | 46.9 (30.8, 67.7) | 54.8 (28.5, 85.4) | 48.8 (28.5, 82.1) | 0.776 |
| Sperm vitality (%); median (P25-P75) | 190 | 81.0 (75.6, 87.0) | 81.5 (73.8, 87.2) | 82.5 (77.5, 90.0) | 0.872 |
| Total motility (%); median (P25-P75) | 191 | 59.5 (44.4, 71.3) | 60.9 (48.9, 71.5) | 69.0 (47.5, 77.0) | 0.477 |
| Progressive motility (%); median (P25-P75) | 191 | 43.3 (29.6, 54.9) | 45.3 (32.1, 57.5) | 47.1 (30.8, 60.0) | 0.722 |
| Normal sperm morphology (%); median (P25-P75) | 190 | 8.8 (4.5, 18.4) | 7.5 (4.5, 14.6) | 11.2 (6.8, 15.1) | 0.404 |
| Total sperm count <39×10^6^ spz.; % (95% CI) | 191 | 4.0 (1.1, 13.5) | 9.8 (5.2, 17.6) | 10.2 (4.4, 21.8) | 0.440 |
| Sperm concentration <15×10^6^ spz./mL; % (95% CI) | 191 | 10.0 (4.3, 21.4) | 8.7 (4.5, 16.2) | 12.2 (5.7, 24.2) | 0.799 |
| Sperm vitality <58%; % (95% CI) | 190 | 6.0 (2.1, 16.2) | 4.4 (1.7, 10.8) | 4.1 (1.1, 13.7) | 0.909 |
| Total motility <40% motile; % (95% CI) | 191 | 12.0 (5.6, 23.8) | 8.7 (4.5, 16.2) | 16.3 (8.5, 29.0) | 0.398 |
| Progressive motility <32% motile; % (95% CI) | 191 | 32.0 (20.8, 45.8) | 25.0 (17.3, 34.7) | 26.5 (16.2, 40.3) | 0.665 |
| Normal sperm morphology <4% normal; % (95% CI) | 190 | 18.0 (9.8, 30.8) | 15.2 (9.3, 23.9) | 4.2 (1.2, 14.0)^a^ | 0.078 |
| Altered seminogram^c^; % (95% CI) | 189 | 50.0 (36.6, 63.4) | 35.2 (26.1, 45.4) | 39.6 (27.0, 53.7) | 0.227 |
| **Polymorphism** |  |  | | | |
| *MTHFR* 677 C>T; % (95% CI) | 191 |  | | | |
| CC | 85 | 46.0 (33.0, 59.6) | 39.1 (29.8, 49.3) | 53.1 (39.4, 66.3) | 0.450 |
| CT | 81 | 40.0 (27.6, 53.8) | 48.9 (38.9, 59.0) | 32.7 (21.2, 46.6) |  |
| TT | 25 | 14.0 (7.0, 26.2) | 12.0 (6.8, 20.2) | 14.3 (7.1, 26.7) |  |
| *MTHFD1* 1958 G>A; % (95% CI) | 191 |  | | | |
| GG | 70 | 34.0 (22.4, 47.8) | 32.6 (23.9, 42.7) | 46.9 (33.7, 60.6) | 0.142 |
| GA | 94 | 44.0 (31.2, 57.7) | 53.3 (43.1, 63.1) | 46.9 (33.7, 60.6) |  |
| AA | 27 | 22.0 (12.8, 35.2) | 14.1 (8.4, 22.7) | 6.1 (2.1, 16.5) |  |
| *MTRR* 524 C>T; % (95% CI) | 191 |  | | | |
| CC | 94 | 50.0 (36.6, 63.4) | 48.9 (38.9, 59.0) | 49.0 (35.6, 62.5) | 0.995 |
| CT | 73 | 38.0 (25.9, 51.8) | 39.1 (29.8, 49.3) | 36.7 (24.7, 50.7) |  |
| TT | 24 | 12.0 (5.6, 23.8) | 12.0 (6.8, 20.2) | 14.3 (7.1, 26.7) |  |
| *MTRR* 66 A>G; % (95% CI) | 190 |  | | | |
| AA | 55 | 32.7 (21.2, 46.6) | 28.3 (20.1, 38.2) | 26.5 (16.2, 40.3) | 0.261 |
| AG | 85 | 53.1 (39.4, 66.3) | 40.2 (30.8, 50.4) | 44.9 (31.9, 58.7) |  |
| GG | 50 | 14.3 (7.1, 26.7) | 31.5 (22.9, 41.6) | 28.6 (17.8, 42.4) |  |
| *TCII* 776 C>G; % (95% CI) | 191 |  | | | |
| CC | 62 | 30.0 (19.1, 43.8) | 32.6 (23.9, 42.7) | 34.7 (22.9, 48.7) | 0.467 |
| CG | 86 | 44.0 (31.2, 57.7) | 50.0 (40.0, 60.0) | 36.7 (24.7, 50.7) |  |
| GG | 43 | 26.0 (15.9, 39.6) | 17.4 (11.0, 26.4) | 28.6 (17.8, 42.4) |  |

Continuous variables are reported as median (25^th^, 75^th^ percentile) and categorical variables as percentage (95% confidence interval). *P*-value was calculated by Kruskal-Wallis test for continuous variables and then corrected for Bonferroni correction for multiple comparisons. Chi-square test was used to compare categorical variables. ^a^*P*<0.05 for comparison of specific genotype versus normal homozygote. ^b^*P*<0.05 for comparison of heterozygote versus variant homozygote. ^c^Participants who have one or more altered sperm quality parameters according to WHO 2010 thresholds.

Abbreviations: %, percentage; BMI, body mass index; MET, metabolic equivalent of task; min, minute; MTHFD1, methylenetetrahydrofolate dehydrogenase-5,10-methylenetetrahydrofolate cyclohydrolase-10-formyltetrahydrofolate synthetase; MTHFR, methylenetetrahydrofolate reductase; MTRR, methionine transferase reductase; n, number of subjects; RBCF, red blood cell folate; SLC19A1, solute carrier family 19 (folate transporter) member 1; TCII, transcobalamin II; tHcy, fasting total homocysteine.

**Supplementary Table S3.** Baseline characteristics of the study population according to *MTHFD1* 1958 G>A polymorphism.

|  | ***MTHFD1* 1958 G>A polymorphism** | | | | |
| --- | --- | --- | --- | --- | --- |
|  | **N** | **GG** | **GA** | **AA** | ***P*-value** |
|  |  | **n = 72** | **n = 95** | **n = 28** |  |
| **Demographic parameters** |  |  |  |  |  |
| Age (years); median (P25-P75) | 195 | 29.0 (25.0, 33.0) | 28.0 (23.0, 32.0) | 26.0 (24.0, 32.0) | 0.154 |
| BMI (kg/m^2^); median (P25-P75) | 195 | 24.4 (22.6, 25.7) | 24.4 (21.8, 26.1) | 23.9 (22.0, 25.5) | 0.464 |
| Physical activity (MET min/week); median (P25-P75) | 195 | 3516 (1982, 5117) | 3511 (1689, 5223) | 4164 (1893, 7078) | 0.700 |
| Sexual abstinence (days); median (P25-P75) | 195 | 4.0 (3.0, 5.0) | 4.0 (3.0, 4.0) | 4.0 (3.0, 6.0) | 0.163 |
| Smoking status; % (95% CI) | 183 |  | | | |
| Non-smoker | 134 | 74.6 (63.1, 83.5) | 70.8 (60.6, 79.2) | 77.8 (59.2, 89.4) | 0.205 |
| Current smoker | 24 | 7.5 (3.2, 16.3) | 15.7 (9.6, 24.7) | 18.5 (8.2, 36.7) |  |
| Former smoker | 25 | 17.9 (10.6, 28.7) | 13.5 (7.9, 22.1) | 3.7 (0.7, 18.3) |  |
| Education; % (95% CI) | 195 |  | | | |
| High school or less | 70 | 41.7 (31.0, 53.2) | 29.5 (21.2, 39.3) | 42.9 (26.5, 60.9) | 0.189 |
| College or high education | 125 | 58.3 (46.8, 69.0) | 70.5 (60.7, 78.8) | 57.1 (39.1, 73.5) |  |
| **Blood parameters** |  |  |  |  |  |
| Creatinine (µmol/L); median (P25-P75) | 194 | 80.5 (75.2, 88.4) | 83.1 (75.2, 90.0) | 83.1 (76.5, 89.1) | 0.501 |
| Plasma folate (nmol/L); median (P25-P75) | 195 | 15.5 (12.3, 18.8) | 13.0 (10.2, 16.6)^a^ | 14.7 (11.0, 19.9) | 0.105 |
| RBCF (nmol/L); median (P25-P75) | 194 | 685.8 (612.5, 788.1) | 676.7 (539.9, 785.7) | 689.7 (572.4, 769.8) | 0.771 |
| Plasma B_1_**_2_** (pmol/L); median (P25-P75) | 195 | 241.6 (205.4, 279.3) | 236.2 (207.6, 287.7) | 254.1 (191.2, 284.2) | 0.662 |
| Plasma tHcy (µmol/L); median (P25-P75) | 195 | 9.7 (7.9, 11.5) | 9.8 (8.1, 11.9) | 9.2 (7.4, 10.4) | 0.249 |
| Plasma folate <7 nmol/L; % (95% CI) | 195 | 1.4 (0.2, 7.5) | 6.3 (2.9, 13.1) | 7.1 (2.0, 22.6) | 0.222 |
| RBCF <340 nmol/L; % (95% CI) | 194 | 1.4 (0.2, 7.5) | 2.1 (0.6, 7.4) | 7.4 (2.1, 23.4) | 0.273 |
| Plasma tHcy >15 µmol/L; % (95% CI) | 195 | 4.2 (1.4, 11.5) | 7.4 (3.6, 14.4) | 3.6 (0.6, 17.7) | 0.692 |
| **Sperm parameters** |  |  |  |  |  |
| Total sperm count (x10^6^ spz.); median (P25-P75) | 195 | 180.5 (92.3, 284.8) | 170.5 (99.8, 289.6) | 135.7 (98.6, 248.5) | 0.249 |
| Sperm concentration (x10^6^ spz./mL); median (P25-P75) | 195 | 53.5 (31.5, 85.3) | 51.1 (30.0, 87.3) | 33.2 (22.7, 56.8)^a^ | 0.125 |
| Sperm vitality (%); median (P25-P75) | 194 | 82.5 (76.0, 89.2) | 80.0 (73.8, 88.0) | 84.2 (80.0, 87.5) | 0.324 |
| Total motility (%); median (P25-P75) | 195 | 64.3 (50.2, 74.6) | 62.0 (47.3, 73.1) | 51.1 (44.1, 62.7) | 0.108 |
| Progressive motility (%); median (P25-P75) | 195 | 48.4 (35.8, 57.5) | 44.5 (30.7, 57.0) | 37.1 (28.2, 49.8) | 0.073 |
| Normal sperm morphology (%); median (P25-P75) | 194 | 10.0 (5.0, 18.2) | 8.5 (5.1, 15.0) | 7.2 (3.9, 12.9) | 0.746 |
| Total sperm count <39×10^6^ spz.; % (95% CI) | 195 | 5.6 (2.2, 13.4) | 9.5 (5.1, 17.0) | 10.7 (3.7, 27.2) | 0.593 |
| Sperm concentration <15×10^6^ spz./mL; % (95% CI) | 195 | 8.3 (3.9, 17.0) | 11.6 (6.6, 19.6) | 7.1 (2.0, 22.6) | 0.753 |
| Sperm vitality <58%; % (95% CI) | 194 | 2.8 (0.8, 9.7) | 7.4 (3.6, 14.4) | 0.0 (0.0, 12.1) | 0.312 |
| Total motility <40% motile; % (95% CI) | 195 | 6.9 (3.0, 15.2) | 13.7 (8.2, 22.0) | 14.3 (5.7, 31.5) | 0.299 |
| Progressive motility <32% motile; % (95% CI) | 195 | 19.4 (12.0, 30.0) | 28.4 (20.3, 38.2) | 42.9 (26.5, 60.9)^a^ | 0.057 |
| Normal sperm morphology <4% normal; % (95% CI) | 194 | 11.3 (5.8, 20.7) | 12.6 (7.4, 20.8) | 25.0 (12.7, 43.4) | 0.181 |
| Altered seminogram^c^; % (95% CI) | 193 | 37.1 (26.8, 48.9) | 35.8 (26.9, 45.8) | 64.3 (45.8, 79.3)^a, b^ | 0.020 |
| **Polymorphism** |  |  | | | |
| *MTHFR* 677 C>T; % (95% CI) | 195 |  | | | |
| CC | 86 | 45.8 (34.8, 57.3) | 41.1 (31.7, 51.1) | 50.0 (32.6, 67.4) | 0.864 |
| CT | 83 | 43.1 (32.3, 54.6) | 44.2 (34.6, 54.2) | 35.7 (20.7, 54.2) |  |
| TT | 26 | 11.1 (5.7, 20.4) | 14.7 (9.0, 23.2) | 14.3 (5.7, 31.5) |  |
| *SLC19A1* 80 G>A; % (95% CI) | 191 |  | | | |
| GG | 50 | 24.3 (15.8, 35.5) | 23.4 (16.0, 32.9) | 40.7 (24.5, 59.3) | 0.142 |
| GA | 92 | 42.9 (31.9, 54.5) | 52.1 (42.1, 61.9) | 48.1 (30.7, 66.0) |  |
| AA | 49 | 32.9 (23.0, 44.5) | 24.5 (16.9, 34.0) | 11.1 (3.9, 28.1) |  |
| *MTRR* 524 C>T; % (95% CI) | 195 |  | | | |
| CC | 96 | 55.6 (44.1, 66.5) | 46.3 (36.6, 56.3) | 42.9 (26.5, 60.9) | 0.216 |
| CT | 74 | 31.9 (22.3, 43.4) | 37.9 (28.8, 47.9) | 53.6 (35.8, 70.5) |  |
| TT | 25 | 12.5 (6.7, 22.1) | 15.8 (9.8, 24.4) | 3.6 (0.6, 17.7) |  |
| *MTRR* 66 A>G; % (95% CI) | 194 |  | | | |
| AA | 56 | 22.5 (14.4, 33.5) | 31.6 (23.1, 41.5) | 35.7 (20.7, 54.2) | 0.534 |
| AG | 86 | 50.7 (39.3, 62.0) | 40.0 (30.7, 50.1) | 42.9 (26.5, 60.9) |  |
| GG | 52 | 26.8 (17.9, 38.1) | 28.4 (20.3, 38.2) | 21.4 (10.2, 39.5) |  |
| *TCII* 776 C>G; % (95% CI) | 195 |  | | | |
| CC | 64 | 41.7 (31.0, 53.2) | 29.5 (21.2, 39.3) | 21.4 (10.2, 39.5) | 0.262 |
| CG | 88 | 37.5 (27.2, 49.0) | 49.5 (39.6, 59.4) | 50.0 (32.6, 67.4) |  |
| GG | 43 | 20.8 (13.1, 31.6) | 21.1 (14.1, 30.3) | 28.6 (15.3, 47.1) |  |

Continuous variables are reported as median (25^th^, 75^th^ percentile) and categorical variables as percentage (95% confidence interval). *P*-value was calculated by Kruskal-Wallis test for continuous variables and then corrected for Bonferroni correction for multiple comparisons. Chi-square test was used to compare categorical variables. ^a^*P*<0.05 for comparison of specific genotype versus normal homozygote. ^b^*P*<0.05 for comparison of heterozygote versus variant homozygote. ^c^Participants who have one or more altered sperm quality parameters according to WHO 2010 thresholds.

Abbreviations: %, percentage; BMI, body mass index; MET, metabolic equivalent of task; min, minute; MTHFD1, methylenetetrahydrofolate dehydrogenase-5,10-methylenetetrahydrofolate cyclohydrolase-10-formyltetrahydrofolate synthetase; MTHFR, methylenetetrahydrofolate reductase; MTRR, methionine transferase reductase; n, number of subjects; RBCF, red blood cell folate; SLC19A1, solute carrier family 19 (folate transporter) member 1; TCII, transcobalamin II; tHcy, fasting total homocysteine.

**Supplementary Table S4.** Baseline characteristics of the study population according to *MTRR* 524 C>T polymorphism.

|  | ***MTRR* 524 C>T polymorphism** | | | | |
| --- | --- | --- | --- | --- | --- |
|  | **N** | **CC** | **CT** | **TT** | ***P*-value** |
|  |  | **n = 96** | **n = 74** | **n = 25** |  |
| **Demographic parameters** |  |  |  |  |  |
| Age (years); median (P25-P75) | 195 | 29.0 (24.8, 33.0) | 26.0 (24.0, 30.8) | 29.0 (24.0, 32.0) | 0.222 |
| BMI (kg/m^2^); median (P25-P75) | 195 | 24.0 (21.9, 25.7) | 24.7 (22.6, 26.3) | 24.6 (22.8, 26.0) | 0.392 |
| Physical activity (MET min/week); median (P25-P75) | 195 | 3647 (1675, 5365) | 3468 (1855, 5435) | 3511 (2551, 4200) | 0.812 |
| Sexual abstinence (days); median (P25-P75) | 195 | 4.0 (3.0, 5.0) | 4.0 (3.0, 5.0) | 4.0 (3.0, 4.0) | 0.798 |
| Smoking status; % (95% CI) | 183 |  | | | |
| Non-smoker | 134 | 72.7 (62.6, 80.9) | 70.0 (58.5, 79.5) | 84.0 (65.3, 93.6) | 0.719 |
| Current smoker | 24 | 14.8 (8.8, 23.7) | 12.9 (6.9, 22.7) | 8.0 (2.2, 25.0) |  |
| Former smoker | 25 | 12.5 (7.1, 21.0) | 17.1 (10.1, 27.6) | 8.0 (2.2, 25.0) |  |
| Education; % (95% CI) | 195 |  | | | |
| High school or less | 70 | 35.4 (26.6, 45.4) | 39.2 (28.9, 50.6) | 28.0 (14.3, 47.6) | 0.596 |
| College or high education | 125 | 64.6 (54.6, 73.4) | 60.8 (49.4, 71.1) | 72.0 (52.4, 85.7) |  |
| **Blood parameters** |  |  |  |  |  |
| Creatinine (µmol/L); median (P25-P75) | 194 | 80.9 (74.3, 87.5) | 83.1 (76.9, 90.2) | 85.8 (77.8, 94.6) | 0.103 |
| Plasma folate (nmol/L); median (P25-P75) | 195 | 14.7 (11.1, 19.4) | 13.3 (10.6, 16.9) | 13.9 (11.7, 17.1) | 0.325 |
| RBCF (nmol/L); median (P25-P75) | 194 | 687.4 (579.5, 837.2) | 665.5 (555.4, 741.2) | 679.0 (645.4, 803.2) | 0.493 |
| Plasma B_1_**_2_** (pmol/L); median (P25-P75) | 195 | 243.7 (205.4, 282.6) | 234.0 (199.1, 279.7) | 242.5 (208.7, 256.3) | 0.753 |
| Plasma tHcy (µmol/L); median (P25-P75) | 195 | 9.3 (7.4, 10.8) | 10.0 (8.3, 11.5) | 9.8 (7.9, 13.8) | 0.554 |
| Plasma folate <7 nmol/L; % (95% CI) | 195 | 5.2 (2.2, 11.6) | 4.1 (1.4, 11.3) | 4.0 (0.7, 19.5) | 0.927 |
| RBCF <340 nmol/L; % (95% CI) | 194 | 2.1 (0.6, 7.4) | 2.7 (0.7, 9.3) | 4.0 (0.7, 19.5) | 0.828 |
| Plasma B_1_**_2_** <148 pmol/L; % (95% CI) | 195 | 1.0 (0.2, 5.7) | 1.4 (0.2, 7.3) | 4.0 (0.7, 19.5) | 0.510 |
| Plasma tHcy >15 µmol/L; % (95% CI) | 195 | 6.3 (2.9, 13.0) | 1.4 (0.2, 7.3) | 16.0 (6.4, 34.7)^b^ | 0.022 |
| **Sperm parameters** |  |  |  |  |  |
| Total sperm count (x10^6^ spz.); median (P25-P75) | 195 | 155.3 (98.6, 289.2) | 180.9 (84.2, 297.7) | 142.8 (117.7, 233.2) | 0.937 |
| Sperm concentration (x10^6^ spz./mL); median (P25-P75) | 195 | 51.8 (31.0, 85.4) | 47.1 (28.6, 81.2) | 41.8 (31.0, 74.2) | 0.393 |
| Sperm vitality (%); median (P25-P75) | 194 | 82.5 (76.2, 88.2) | 81.0 (76.0, 87.4) | 85.0 (72.0, 91.0) | 0.377 |
| Total motility (%); median (P25-P75) | 195 | 61.2 (47.4, 72.5) | 64.1 (49.0, 74.6) | 51.6 (46.9, 72.9) | 0.737 |
| Progressive motility (%); median (P25-P75) | 195 | 43.6 (32.6, 55.1) | 47.5 (31.1, 57.5) | 38.7 (28.2, 52.4) | 0.430 |
| Normal sperm morphology (%); median (P25-P75) | 194 | 8.0 (4.8, 15.2) | 8.2 (5.0, 14.8) | 10.5 (5.5, 18.5) | 0.323 |
| Total sperm count <39×10^6^ spz.; % (95% CI) | 195 | 9.4 (5.0, 16.9) | 5.4 (2.1, 13.1) | 12.0 (4.2, 30.0) | 0.433 |
| Sperm concentration <15×10^6^ spz./mL; % (95% CI) | 195 | 10.4 (5.8, 18.1) | 8.1 (3.8, 16.6) | 12.0 (4.2, 30.0) | 0.794 |
| Sperm vitality <58%; % (95% CI) | 194 | 5.3 (2.3, 11.7) | 2.7 (0.7, 9.3) | 8.0 (2.2, 25.0) | 0.343 |
| Total motility <40% motile; % (95% CI) | 195 | 11.5 (6.5, 19.4) | 10.8 (5.6, 19.9) | 12.0 (4.2, 30.0) | 1.000 |
| Progressive motility <32% motile; % (95% CI) | 195 | 24.0 (16.5, 33.4) | 27.0 (18.2, 38.1) | 40.0 (23.4, 59.3) | 0.275 |
| Normal sperm morphology <4% normal; % (95% CI) | 194 | 13.7 (8.2, 22.0) | 16.2 (9.5, 26.2) | 8.0 (2.2, 25.0) | 0.678 |
| Altered seminogram^c^; % (95% CI) | 193 | 38.3 (29.1, 48.4) | 39.2 (28.9, 50.6) | 52.0 (33.5, 70.0) | 0.446 |
| **Polymorphism** |  |  | | | |
| *MTHFR* 677 C>T; % (95% CI) | 195 |  | | | |
| CC | 86 | 44.8 (35.2, 54.7) | 41.9 (31.3, 53.3) | 48.0 (30.0, 66.5) | 0.960 |
| CT | 83 | 40.6 (31.3, 50.6) | 45.9 (35.1, 57.2) | 40.0 (23.4, 59.3) |  |
| TT | 26 | 14.6 (8.9, 23.0) | 12.2 (6.5, 21.5) | 12.0 (4.2, 30.0) |  |
| *SLC19A1* 80 G>A; % (95% CI) | 191 |  | | | |
| GG | 50 | 26.6 (18.7, 36.3) | 26.0 (17.3, 37.1) | 25.0 (12.0, 44.9) | 0.995 |
| GA | 92 | 47.9 (38.1, 57.9) | 49.3 (38.2, 60.5) | 45.8 (27.9, 64.9) |  |
| AA | 49 | 25.5 (17.8, 35.2) | 24.7 (16.2, 35.6) | 29.2 (14.9, 49.2) |  |
| *MTHFD1* 1958 G>A; % (95% CI) | 195 |  | | | |
| GG | 72 | 41.7 (32.3, 51.7) | 31.1 (21.7, 42.3) | 36.0 (20.2, 55.5) | 0.216 |
| GA | 95 | 45.8 (36.2, 55.8) | 48.6 (37.6, 59.8) | 60.0 (40.7, 76.6) |  |
| AA | 28 | 12.5 (7.3, 20.6) | 20.3 (12.7, 30.8) | 4.0 (0.7, 19.5) |  |
| *MTRR* 66 A>G; % (95% CI) | 194 |  | | | |
| AA | 56 | 33.3 (24.7, 43.2) | 30.1 (20.8, 41.4) | 8.0 (2.2, 25.0) | 0.040 |
| AG | 86 | 41.7 (32.3, 51.7) | 47.9 (36.9, 59.2) | 44.0 (26.7, 62.9) |  |
| GG | 52 | 25.0 (17.4, 34.5) | 21.9 (14.0, 32.7) | 48.0 (30.0, 66.5) |  |
| *TCII* 776 C>G; % (95% CI) | 195 |  | | | |
| CC | 64 | 38.5 (29.4, 48.5) | 27.0 (18.2, 38.1) | 28.0 (14.3, 47.6) | 0.513 |
| CG | 88 | 42.7 (33.3, 52.7) | 48.6 (37.6, 59.8) | 44.0 (26.7, 62.9) |  |
| GG | 43 | 18.8 (12.2, 27.7) | 24.3 (16.0, 35.2) | 28.0 (14.3, 47.6) |  |

Continuous variables are reported as median (25^th^, 75^th^ percentile) and categorical variables as percentage (95% confidence interval). *P*-value was calculated by Kruskal-Wallis test for continuous variables and then corrected for Bonferroni correction for multiple comparisons. Chi-square test was used to compare categorical variables. ^a^*P*<0.05 for comparison of specific genotype versus normal homozygote. ^b^*P*<0.05 for comparison of heterozygote versus variant homozygote. ^c^Participants who have one or more altered sperm quality parameters according to WHO 2010 thresholds.

Abbreviations: %, percentage; BMI, body mass index; MET, metabolic equivalent of task; min, minute; MTHFD1, methylenetetrahydrofolate dehydrogenase-5,10-methylenetetrahydrofolate cyclohydrolase-10-formyltetrahydrofolate synthetase; MTHFR, methylenetetrahydrofolate reductase; MTRR, methionine transferase reductase; n, number of subjects; RBCF, red blood cell folate; SLC19A1, solute carrier family 19 (folate transporter) member 1; TCII, transcobalamin II; tHcy, fasting total homocysteine.

**Supplementary Table S5.** Baseline characteristics of the study population according to *MTRR* 66 A>G polymorphism.

|  | ***MTRR* 66 A>G polymorphism** | | | | |
| --- | --- | --- | --- | --- | --- |
|  | **N** | **AA** | **AG** | **GG** | ***P*-value** |
|  |  | **n = 56** | **n = 86** | **n = 52** |  |
| **Demographic parameters** |  |  |  |  |  |
| Age (years); median (P25-P75) | 194 | 29.0 (23.8, 33.0) | 28.0 (24.0, 32.0) | 28.5 (24.0, 32.2) | 0.943 |
| BMI (kg/m^2^); median (P25-P75) | 194 | 24.6 (22.0, 26.2) | 24.3 (21.9, 25.8) | 24.4 (22.7, 25.9) | 0.847 |
| Physical activity (MET min/week); median (P25-P75) | 194 | 3495 (1662, 4934) | 3693 (1877, 5882) | 3497 (1962, 5115) | 0.943 |
| Sexual abstinence (days); median (P25-P75) | 194 | 4.0 (3.0, 5.0) | 4.0 (3.0, 5.0) | 4.0 (3.0, 4.1) | 0.439 |
| Smoking status; % (95% CI) | 182 |  | | | |
| Non-smoker | 133 | 62.3 (48.8, 74.1) | 75.9 (65.7, 83.8) | 80.4 (66.8, 89.3) | 0.283 |
| Current smoker | 24 | 17.0 (9.2, 29.2) | 13.3 (7.6, 22.2) | 8.7 (3.4, 20.3) |  |
| Former smoker | 25 | 20.8 (12.0, 33.5) | 10.8 (5.8, 19.3) | 10.9 (4.7, 23.0) |  |
| Education; % (95% CI) | 194 |  | | | |
| High school or less | 69 | 37.5 (26.0, 50.6) | 33.7 (24.6, 44.2) | 36.5 (24.8, 50.1) | 0.887 |
| College or high education | 125 | 62.5 (49.4, 74.0) | 66.3 (55.8, 75.4) | 63.5 (49.9, 75.2) |  |
| **Blood parameters** |  |  |  |  |  |
| Creatinine (µmol/L); median (P25-P75) | 193 | 82.2 (74.3, 88.6) | 83.1 (75.2, 90.0) | 82.2 (75.6, 88.4) | 0.828 |
| Plasma folate (nmol/L); median (P25-P75) | 194 | 14.3 (10.9, 17.6) | 14.5 (11.5, 18.9) | 13.3 (9.9, 17.3) | 0.429 |
| RBCF (nmol/L); median (P25-P75) | 193 | 704.4 (587.6, 778.7) | 665.3 (589.1, 803.3) | 676.8 (574.0, 776.2) | 0.247 |
| Plasma B_1_**_2_** (pmol/L); median (P25-P75) | 194 | 232.9 (198.9, 264.6) | 252.5 (213.9, 299.1)^a^ | 230.1 (194.6, 276.7) | 0.065 |
| Plasma tHcy (µmol/L); median (P25-P75) | 194 | 9.1 (7.6, 11.2) | 9.7 (7.9, 11.6) | 9.8 (7.9, 11.8) | 0.609 |
| Plasma folate <7 nmol/L; % (95% CI) | 194 | 1.8 (0.3, 9.4) | 5.8 (2.5, 12.9) | 5.8 (2.0, 15.6) | 0.568 |
| RBCF <340 nmol/L; % (95% CI) | 193 | 1.8 (0.3, 9.4) | 2.4 (0.6, 8.2) | 3.8 (1.1, 13.0) | 0.729 |
| Plasma B_1_**_2_** <148 pmol/L; % (95% CI) | 194 | 1.8 (0.3, 9.4) | 1.2 (0.2, 6.3) | 1.9 (0.3, 10.1) | 1.000 |
| Plasma tHcy >15 µmol/L; % (95% CI) | 194 | 5.4 (1.8, 14.6) | 5.8 (2.5, 12.9) | 5.8 (2.0, 15.6) | 1.000 |
| **Sperm parameters** |  |  |  |  |  |
| Total sperm count (x10^6^ spz.); median (P25-P75) | 194 | 142.1 (100.7, 296.0) | 172.5 (85.3, 283.2) | 150.6 (106.0, 272.0) | 0.678 |
| Sperm concentration (x10^6^ spz./mL); median (P25-P75) | 194 | 51.2 (28.4, 87.4) | 48.2 (30.6, 79.2) | 50.2 (29.8, 84.7) | 0.678 |
| Sperm vitality (%); median (P25-P75) | 193 | 80.2 (73.8, 85.0) | 82.5 (76.0, 91.0) | 81.8 (76.5, 87.1) | 0.629 |
| Total motility (%); median (P25-P75) | 194 | 60.4 (47.1, 72.6) | 60.9 (46.9, 74.0) | 61.9 (49.6, 72.4) | 0.623 |
| Progressive motility (%); median (P25-P75) | 194 | 45.2 (31.2, 57.5) | 45.3 (29.7, 56.3) | 45.5 (32.7, 53.4) | 0.902 |
| Normal sperm morphology (%); median (P25-P75) | 193 | 8.2 (5.0, 15.1) | 9.5 (5.0, 15.0) | 7.0 (4.5, 15.1) | 0.717 |
| Total sperm count <39×10^6^ spz.; % (95% CI) | 194 | 12.5 (6.2, 23.6) | 8.1 (4.0, 15.9) | 3.8 (1.1, 13.0) | 0.251 |
| Sperm concentration <15×10^6^ spz./mL; % (95% CI) | 194 | 12.5 (6.2, 23.6) | 10.5 (5.6, 18.7) | 5.8 (2.0, 15.6) | 0.505 |
| Sperm vitality <58%; % (95% CI) | 193 | 5.4 (1.8, 14.6) | 3.5 (1.2, 9.9) | 5.8 (2.0, 15.6) | 0.752 |
| Total motility <40% motile; % (95% CI) | 194 | 12.5 (6.2, 23.6) | 10.5 (5.6, 18.7) | 11.5 (5.4, 23.0) | 0.931 |
| Progressive motility <32% motile; % (95% CI) | 194 | 26.8 (17.0, 39.6) | 29.1 (20.5, 39.4) | 25.0 (15.2, 38.2) | 0.869 |
| Normal sperm morphology <4% normal; % (95% CI) | 193 | 16.1 (8.7, 27.8) | 11.8 (6.5, 20.3) | 15.4 (8.0, 27.5) | 0.728 |
| Altered seminogram^c^; % (95% CI) | 192 | 35.7 (24.5, 48.8) | 45.2 (35.0, 55.9) | 38.5 (26.5, 52.0) | 0.496 |
| **Polymorphism** |  |  | | | |
| *MTHFR* 677 C>T; % (95% CI) | 194 |  | | | |
| CC | 86 | 51.8 (39.0, 64.3) | 45.3 (35.3, 55.8) | 34.6 (23.2, 48.2) | 0.501 |
| CT | 82 | 35.7 (24.5, 48.8) | 41.9 (32.0, 52.4) | 50.0 (36.9, 63.1) |  |
| TT | 26 | 12.5 (6.2, 23.6) | 12.8 (7.3, 21.5) | 15.4 (8.0, 27.5) |  |
| *SLC19A1* 80 G>A; % (95% CI) | 190 |  | | | |
| GG | 49 | 29.1 (18.8, 42.1) | 30.6 (21.8, 41.0) | 14.0 (7.0, 26.2) | 0.261 |
| GA | 92 | 47.3 (34.7, 60.2) | 43.5 (33.5, 54.1) | 58.0 (44.2, 70.6) |  |
| AA | 49 | 23.6 (14.4, 36.3) | 25.9 (17.8, 36.1) | 28.0 (17.5, 41.7) |  |
| *MTHFD1* 1958 G>A; % (95% CI) | 194 |  | | | |
| GG | 71 | 28.6 (18.4, 41.5) | 41.9 (32.0, 52.4) | 36.5 (24.8, 50.1) | 0.534 |
| GA | 95 | 53.6 (40.7, 66.0) | 44.2 (34.2, 54.7) | 51.9 (38.7, 64.9) |  |
| AA | 28 | 17.9 (10.0, 29.8) | 14.0 (8.2, 22.8) | 11.5 (5.4, 23.0) |  |
| *MTRR* 524 C>T; % (95% CI) | 194 |  | | | |
| CC | 96 | 57.1 (44.1, 69.2) | 46.5 (36.3, 57.0) | 46.2 (33.3, 59.5) | 0.044 |
| CT | 73 | 39.3 (27.6, 52.4) | 40.7 (30.9, 51.3) | 30.8 (19.9, 44.3) |  |
| TT | 25 | 3.6 (1.0, 12.1) | 12.8 (7.3, 21.5) | 23.1 (13.7, 36.1) |  |
| *TCII* 776 C>G; % (95% CI) | 194 |  | | | |
| CC | 64 | 35.7 (24.5, 48.8) | 33.7 (24.6, 44.2) | 28.8 (18.3, 42.3) | 0.731 |
| CG | 87 | 46.4 (34.0, 59.3) | 40.7 (30.9, 51.3) | 50.0 (36.9, 63.1) |  |
| GG | 43 | 17.9 (10.0, 29.8) | 25.6 (17.5, 35.7) | 21.2 (12.2, 34.0) |  |

Continuous variables are reported as median (25^th^, 75^th^ percentile) and categorical variables as percentage (95% confidence interval). *P*-value was calculated by Kruskal-Wallis test for continuous variables and then corrected for Bonferroni correction for multiple comparisons. Chi-square test was used to compare categorical variables. ^a^*P*<0.05 for comparison of specific genotype versus normal homozygote. ^b^*P*<0.05 for comparison of heterozygote versus variant homozygote. ^c^Participants who have one or more altered sperm quality parameters according to WHO 2010 thresholds.

Abbreviations: %, percentage; BMI, body mass index; MET, metabolic equivalent of task; min, minute; MTHFD1, methylenetetrahydrofolate dehydrogenase-5,10-methylenetetrahydrofolate cyclohydrolase-10-formyltetrahydrofolate synthetase; MTHFR, methylenetetrahydrofolate reductase; MTRR, methionine transferase reductase; n, number of subjects; RBCF, red blood cell folate; SLC19A1, solute carrier family 19 (folate transporter) member 1; TCII, transcobalamin II; tHcy, fasting total homocysteine.

**Supplementary Table S6.** Baseline characteristics of the study population according to *TCII* 776 C>G polymorphism.

|  | ***TCII* 776 C>G polymorphism** | | | | |
| --- | --- | --- | --- | --- | --- |
|  | **N** | **CC** | **CG** | **GG** | ***P*-value** |
|  |  | **n = 64** | **n = 88** | **n = 43** |  |
| **Demographic parameters** |  |  |  |  |  |
| Age (years); median (P25-P75) | 195 | 29.0 (24.8, 33.0) | 27.5 (23.0, 32.0) | 29.0 (24.5, 33.5) | 0.251 |
| BMI (kg/m^2^); median (P25-P75) | 195 | 24.8 (22.8, 27.2) | 24.1 (22.2, 25.6) | 23.9 (21.7, 25.7) | 0.420 |
| Physical activity (MET min/week); median (P25-P75) | 195 | 2850 (1683, 5366) | 3647 (1982, 5365) | 3865 (2052, 4984) | 0.315 |
| Sexual abstinence (days); median (P25-P75) | 195 | 4.0 (3.0, 5.0) | 4.0 (3.0, 5.0) | 4.0 (3.0, 5.0) | 0.650 |
| Smoking status; % (95% CI) | 183 |  | | | |
| Non-smoker | 134 | 69.0 (56.2, 79.4) | 75.0 (64.8, 83.0) | 75.6 (60.7, 86.2) | 0.936 |
| Current smoker | 24 | 15.5 (8.4, 26.9) | 11.9 (6.6, 20.5) | 12.2 (5.3, 25.5) |  |
| Former smoker | 25 | 15.5 (8.4, 26.9) | 13.1 (7.5, 21.9) | 12.2 (5.3, 25.5) |  |
| Education; % (95% CI) | 195 |  | | | |
| High school or less | 70 | 43.8 (32.3, 55.9) | 36.4 (27.1, 46.8) | 23.3 (13.2, 37.7) | 0.095 |
| College or high education | 125 | 56.2 (44.1, 67.7) | 63.6 (53.2, 72.9) | 76.7 (62.3, 86.8) |  |
| **Blood parameters** |  |  |  |  |  |
| Creatinine (µmol/L); median (P25-P75) | 194 | 80.5 (74.3, 86.7) | 83.1 (76.5, 89.3) | 84.0 (75.6, 90.2) | 0.266 |
| Plasma folate (nmol/L); median (P25-P75) | 195 | 13.2 (10.6, 17.5) | 14.7 (10.8, 19.1) | 14.7 (11.6, 17.6) | 0.114 |
| RBCF (nmol/L); median (P25-P75) | 194 | 676.5 (562.9, 758.6) | 688.0 (587.0, 804.2) | 682.3 (627.8, 760.1) | 0.659 |
| Plasma B_1_**_2_** (pmol/L); median (P25-P75) | 195 | 237.1 (208.3, 269.9) | 244.2 (197.7, 284.4) | 244.7 (205.6, 298.8) | 0.668 |
| Plasma tHcy (µmol/L); median (P25-P75) | 195 | 10.2 (8.4, 12.0) | 9.2 (7.5, 11.0) | 9.8 (7.9, 11.9) | 0.202 |
| Plasma folate <7 nmol/L; % (95% CI) | 195 | 3.1 (0.9, 10.7) | 6.8 (3.2, 14.1) | 2.3 (0.4, 12.1) | 0.495 |
| RBCF <340 nmol/L; % (95% CI) | 194 | 3.1 (0.9, 10.7) | 2.3 (0.6, 7.9) | 2.4 (0.4, 12.3) | 1.000 |
| Plasma B_1_**_2_** <148 pmol/L; % (95% CI) | 195 | 1.6 (0.3, 8.3) | 2.3 (0.6, 7.9) | 0.0 (0.0, 8.2) | 1.000 |
| Plasma tHcy >15 µmol/L; % (95% CI) | 195 | 1.6 (0.3, 8.3) | 9.1 (4.7, 16.9) | 4.7 (1.3, 15.5) | 0.146 |
| **Sperm parameters** |  |  |  |  |  |
| Total sperm count (x10^6^ spz.); median (P25-P75) | 195 | 157.5 (100.8, 270.9) | 160.3 (94.7, 299.5) | 202.7 (94.6, 272.8) | 0.856 |
| Sperm concentration (x10^6^ spz./mL); median (P25-P75) | 195 | 51.7 (31.6, 86.7) | 47.3 (28.7, 86.7) | 48.6 (27.3, 70.9) | 0.624 |
| Sperm vitality (%); median (P25-P75) | 194 | 81.0 (76.0, 87.2) | 82.8 (75.5, 87.9) | 81.0 (76.2, 88.9) | 0.502 |
| Total motility (%); median (P25-P75) | 195 | 62.8 (45.9, 73.8) | 59.3 (47.1, 71.5) | 68.3 (52.9, 75.0)^b^ | 0.020 |
| Progressive motility (%); median (P25-P75) | 195 | 47.7 (33.1, 58.2) | 43.6 (28.9, 55.5) | 48.9 (36.8, 56.0) | 0.254 |
| Normal sperm morphology (%); median (P25-P75) | 194 | 9.5 (4.8, 16.2) | 9.5 (5.0, 14.6) | 8.5 (5.0, 14.2) | 0.874 |
| Total sperm count <39×10^6^ spz.; % (95% CI) | 195 | 9.4 (4.4, 19.0) | 9.1 (4.7, 16.9) | 4.7 (1.3, 15.5) | 0.708 |
| Sperm concentration <15×10^6^ spz./mL; % (95% CI) | 195 | 7.8 (3.4, 17.0) | 12.5 (7.1, 21.0) | 7.0 (2.4, 18.6) | 0.604 |
| Sperm vitality <58%; % (95% CI) | 194 | 6.2 (2.5, 15.0) | 5.7 (2.5, 12.6) | 0.0 (0.0, 8.4) | 0.278 |
| Total motility <40% motile; % (95% CI) | 195 | 10.9 (5.4, 20.9) | 14.8 (8.8, 23.7) | 4.7 (1.3, 15.5) | 0.251 |
| Progressive motility <32% motile; % (95% CI) | 195 | 21.9 (13.5, 33.4) | 34.1 (25.0, 44.5) | 20.9 (11.4, 35.2) | 0.144 |
| Normal sperm morphology <4% normal; % (95% CI) | 194 | 14.3 (7.7, 25.0) | 13.6 (8.0, 22.3) | 14.0 (6.6, 27.3) | 0.994 |
| Altered seminogram^c^; % (95% CI) | 193 | 39.7 (28.5, 52.0) | 43.2 (33.3, 53.6) | 35.7 (23.0, 50.8) | 0.712 |
| **Polymorphism** |  |  | | | |
| *MTHFR* 677 C>T; % (95% CI) | 195 |  | | | |
| CC | 86 | 45.3 (33.7, 57.4) | 40.9 (31.2, 51.4) | 48.8 (34.6, 63.2) | 0.884 |
| CT | 83 | 40.6 (29.5, 52.9) | 44.3 (34.4, 54.7) | 41.9 (28.4, 56.7) |  |
| TT | 26 | 14.1 (7.6, 24.6) | 14.8 (8.8, 23.7) | 9.3 (3.7, 21.6) |  |
| *SLC19A1* 80 G>A; % (95% CI) | 191 |  | | | |
| GG | 50 | 24.2 (15.2, 36.2) | 25.6 (17.5, 35.7) | 30.2 (18.6, 45.1) | 0.467 |
| GA | 92 | 48.4 (36.4, 60.6) | 53.5 (43.0, 63.7) | 37.2 (24.4, 52.1) |  |
| AA | 49 | 27.4 (17.9, 39.6) | 20.9 (13.7, 30.7) | 32.6 (20.5, 47.5) |  |
| *MTHFD1* 1958 G>A; % (95% CI) | 195 |  | | | |
| GG | 72 | 46.9 (35.2, 58.9) | 30.7 (22.0, 41.0) | 34.9 (22.4, 49.8) | 0.262 |
| GA | 95 | 43.8 (32.3, 55.9) | 53.4 (43.1, 63.5) | 46.5 (32.5, 61.1) |  |
| AA | 28 | 9.4 (4.4, 19.0) | 15.9 (9.7, 25.0) | 18.6 (9.7, 32.6) |  |
| *MTRR* 524 C>T; % (95% CI) | 195 |  | | | |
| CC | 96 | 57.8 (45.6, 69.1) | 46.6 (36.5, 56.9) | 41.9 (28.4, 56.7) | 0.513 |
| CT | 74 | 31.2 (21.2, 43.4) | 40.9 (31.2, 51.4) | 41.9 (28.4, 56.7) |  |
| TT | 25 | 10.9 (5.4, 20.9) | 12.5 (7.1, 21.0) | 16.3 (8.1, 30.0) |  |
| *MTRR* 66 A>G; % (95% CI) | 194 |  | | | |
| AA | 56 | 31.2 (21.2, 43.4) | 29.9 (21.3, 40.2) | 23.3 (13.2, 37.7) | 0.731 |
| AG | 86 | 45.3 (33.7, 57.4) | 40.2 (30.6, 50.7) | 51.2 (36.8, 65.4) |  |
| GG | 52 | 23.4 (14.7, 35.1) | 29.9 (21.3, 40.2) | 25.6 (14.9, 40.2) |  |

Continuous variables are reported as median (25^th^, 75^th^ percentile) and categorical variables as percentage (95% confidence interval). *P*-value was calculated by Kruskal-Wallis test for continuous variables and then corrected for Bonferroni correction for multiple comparisons. Chi-square test was used to compare categorical variables. ^a^*P*<0.05 for comparison of specific genotype versus normal homozygote. ^b^*P*<0.05 for comparison of heterozygote versus variant homozygote. ^c^Participants who have one or more altered sperm quality parameters according to WHO 2010 thresholds.

Abbreviations: %, percentage; BMI, body mass index; MET, metabolic equivalent of task; min, minute; MTHFD1, methylenetetrahydrofolate dehydrogenase-5,10-methylenetetrahydrofolate cyclohydrolase-10-formyltetrahydrofolate synthetase; MTHFR, methylenetetrahydrofolate reductase; MTRR, methionine transferase reductase; n, number of subjects; RBCF, red blood cell folate; SLC19A1, solute carrier family 19 (folate transporter) member 1; TCII, transcobalamin II; tHcy, fasting total homocysteine.

**Supplementary Table S7.** Multiple linear regression analysis to test the associations between *MTHFR* 677 C>T polymorphism and sperm quality parameters.

| **Sperm quality parameters** | **Categories of *MTHFR* 677 C>T polymorphism** | | | | |
| --- | --- | --- | --- | --- | --- |
|  | **CC** | **CT vs. CC** | **TT vs. CC** | ***P-*value^b^** | **R^2^** |
| n | 86 | 83 | 26 |  |  |
| Total sperm count (x10^6^ spz.)^a^ |  |  |  |  |  |
| Crude model | Ref. | -0.088 (0.154) | 0.028 (0.223) | 0.800 | 0.002 |
| Model 1 | Ref. | -0.115 (0.153) | 0.113 (0.224) | 0.036 | 0.105 |
| Sperm concentration (x10^6^ spz./mL)^a^ |  |  |  |  |  |
| Crude model | Ref. | -0.091 (0.144) | 0.081 (0.209) | 0.666 | 0.004 |
| Model 1 | Ref. | -0.099 (0.146) | 0.181 (0.214) | 0.198 | 0.076 |
| Sperm vitality (%)^a^ |  |  |  |  |  |
| Crude model | Ref. | -0.009 (0.025) | 0.004 (0.037) | 0.911 | 0.001 |
| Model 1 | Ref. | -0.010 (0.026) | 0.016 (0.038) | 0.717 | 0.042 |
| Total motility (%)^a^ |  |  |  |  |  |
| Crude model | Ref. | -0.078 (0.056) | 0.060 (0.081) | 0.166 | 0.019 |
| Model 1 | Ref. | -0.052 (0.056) | 0.122 (0.082) | 0.037 | 0.105 |
| Progressive motility (%) |  |  |  |  |  |
| Crude model | Ref. | -2.853 (2.600) | 1.165 (3.782) | 0.424 | 0.009 |
| Model 1 | Ref. | -1.524 (2.636) | 3.897 (3.854) | 0.100 | 0.089 |
| Normal sperm forms (%)^a^ |  |  |  |  |  |
| Crude model | Ref. | -0.183 (0.120) | 0.012 (0.176) | 0.261 | 0.014 |
| Model 1 | Ref. | -0.180 (0.123) | -0.017 (0.183) | 0.298 | 0.068 |

Values are B non-standardized coefficient and standard errors. ^a^Variable were natural log transformed to approximate to a normal distribution. ^b^Model *P-*value. Crude model: unadjusted. Model 1 adjusted for: plasma folate (nmol/L), plasma cobalamin (pmol/L), age (years), BMI (kg/m^2^), education (high school or less, college or high education), smoking status (non-smoker, current smoker, and former smoker), physical activity (MET min/week), abstinence days (days), and creatinine (µmol/L).

Abbreviations. %, percentage; BMI, body mass index; MET, metabolic equivalent of task; min, minute; MTHFR, methylenetetrahydrofolate reductase; n, number of subjects.

**Supplementary Table S8.** Multiple linear regression analysis to test the associations between *SLC19A1* 80 G>A polymorphism and sperm quality parameters.

| **Sperm quality parameters** | **Categories of *SLC19A1* 80 G>A polymorphism** | | | | |
| --- | --- | --- | --- | --- | --- |
|  | **GG** | **GA vs. GG** | **AA vs. GG** | ***P-*value^b^** | **R^2^** |
| n | 50 | 92 | 49 |  |  |
| Total sperm count (x10^6^ spz.)^a^ |  |  |  |  |  |
| Crude model | Ref. | -0.230 (0.176) | -0.215 (0.201) | 0.396 | 0.010 |
| Model 1 | Ref. | -0.262 (0.173) | -0.290 (0.200) | 0.016 | 0.120 |
| Sperm concentration (x10^6^ spz./mL)^a^ |  |  |  |  |  |
| Crude model | Ref. | -0.042 (0.166) | 0.025 (0.190) | 0.917 | 0.001 |
| Model 1 | Ref. | -0.036 (0.167) | 0.003 (0.193) | 0.215 | 0.076 |
| Sperm vitality (%)^a^ |  |  |  |  |  |
| Crude model | Ref. | 0.008 (0.029) | 0.028 (0.033) | 0.679 | 0.004 |
| Model 1 | Ref. | 0.011 (0.030) | 0.023 (0.034) | 0.673 | 0.046 |
| Total motility (%)^a^ |  |  |  |  |  |
| Crude model | Ref. | -0.005 (0.064) | 0.041 (0.074) | 0.768 | 0.003 |
| Model 1 | Ref. | -0.005 (0.064) | 0.009 (0.074) | 0.096 | 0.091 |
| Progressive motility (%) |  |  |  |  |  |
| Crude model | Ref. | 1.375 (2.990) | 2.762 (3.421) | 0.722 | 0.003 |
| Model 1 | Ref. | 0.869 (2.998) | 1.166 (3.462) | 0.123 | 0.086 |
| Normal sperm forms (%)^a^ |  |  |  |  |  |
| Crude model | Ref. | -0.033 (0.136) | 0.225 (0.157) | 0.164 | 0.019 |
| Model 1 | Ref. | -0.093 (0.137) | 0.208 (0.160) | 0.155 | 0.084 |

Values are B non-standardized coefficient and standard errors. ^a^Variable were natural log transformed to approximate to a normal distribution. ^b^Model *P-*value. Crude model: unadjusted. Model 1 adjusted for: plasma folate (nmol/L), plasma cobalamin (pmol/L), age (years), BMI (kg/m^2^), education (high school or less, college or high education), smoking status (non-smoker, current smoker, and former smoker), physical activity (MET min/week), abstinence days (days), and creatinine (µmol/L).

Abbreviations. %, percentage; BMI, body mass index; MET, metabolic equivalent of task; min, minute; n, number of subjects; SLC19A1, solute carrier family 19 (folate transporter) member 1.

**Supplementary Table S9.** Multiple linear regression analysis to test the associations between *MTHFD1* 1958 G>A polymorphism and sperm quality parameters.

| **Sperm quality parameters** | **Categories of *MTHFD1* 1958 G>A polymorphism** | | | | |
| --- | --- | --- | --- | --- | --- |
|  | **GG** | **GA vs. GG** | **AA vs. GG** | ***P-*value^b^** | **R^2^** |
| n | 72 | 95 | 28 |  |  |
| Total sperm count (x10^6^ spz.)^a^ |  |  |  |  |  |
| Crude model | Ref. | -0.029 (0.156) | -0.211 (0.222) | 0.623 | 0.005 |
| Model 1 | Ref. | 0.049 (0.157) | -0.167 (0.222) | 0.039 | 0.104 |
| Sperm concentration (x10^6^ spz./mL)^a^ |  |  |  |  |  |
| Crude model | Ref. | -0.020 (0.145) | -0.344 (0.207) | 0.215 | 0.016 |
| Model 1 | Ref. | 0.046 (0.149) | -0.276 (0.211) | 0.164 | 0.079 |
| Sperm vitality (%)^a^ |  |  |  |  |  |
| Crude model | Ref. | -0.039 (0.025) | 0.026 (0.036) | 0.105 | 0.023 |
| Model 1 | Ref. | -0.047 (0.026) | 0.017 (0.037) | 0.337 | 0.065 |
| Total motility (%)^a^ |  |  |  |  |  |
| Crude model | Ref. | -0.081 (0.057) | -0.145 (0.081) | 0.148 | 0.020 |
| Model 1 | Ref. | -0.077 (0.058) | -0.129 (0.082) | 0.055 | 0.099 |
| Progressive motility (%) |  |  |  |  |  |
| Crude model | Ref. | -3.855 (2.616) | -8.254 (3.729)^c^ | 0.073 | 0.027 |
| Model 1 | Ref. | -3.080 (2.671) | -7.732 (3.782)^c^ | 0.049 | 0.100 |
| Normal sperm forms (%)^a^ |  |  |  |  |  |
| Crude model | Ref. | -0.098 (0.123) | -0.291 (0.173) | 0.246 | 0.015 |
| Model 1 | Ref. | -0.117 (0.126) | -0.333 (0.177) | 0.221 | 0.075 |

Values are B non-standardized coefficient and standard errors. ^a^Variable were natural log transformed to approximate to a normal distribution. ^b^Model *P-*value. ^c^*P-*value <0.05 for comparison of specific genotype versus normal homozygote genotype (reference). Crude model: unadjusted. Model 1 adjusted for: plasma folate (nmol/L), plasma cobalamin (pmol/L), age (years), BMI (kg/m^2^), education (high school or less, college or high education), smoking status (non-smoker, current smoker, and former smoker), physical activity (MET min/week), abstinence days (days), and creatinine (µmol/L).

Abbreviations. %, percentage; BMI, body mass index; MET, metabolic equivalent of task; min, minute; MTHFD1, methylenetetrahydrofolate dehydrogenase-5,10-methylenetetrahydrofolate cyclohydrolase-10-formyltetrahydrofolate synthetase; n, number of subjects.

**Supplementary Table S10.** Multiple linear regression analysis to test the associations between *MTRR* 524 C>T polymorphism and sperm quality parameters.

| **Sperm quality parameters** | **Categories of *MTRR* 524 C>T polymorphism** | | | | |
| --- | --- | --- | --- | --- | --- |
|  | **CC** | **CT vs. CC** | **TT vs. CC** | ***P-*value^b^** | **R^2^** |
| n | 96 | 74 | 25 |  |  |
| Total sperm count (x10^6^ spz.)^a^ |  |  |  |  |  |
| Crude model | Ref. | 0.001 (0.155) | -0.027 (0.224) | 0.992 | 0.001 |
| Model 1 | Ref. | 0.089 (0.156) | 0.073 (0.223) | **0.047** | 0.101 |
| Sperm concentration (x10^6^ spz./mL)^a^ |  |  |  |  |  |
| Crude model | Ref. | -0.082 (0.145) | -0.030 (0.210) | 0.853 | 0.002 |
| Model 1 | Ref. | -0.004 (0.149) | 0.021 (0.213) | 0.299 | 0.067 |
| Sperm vitality (%)^a^ |  |  |  |  |  |
| Crude model | Ref. | -0.013 (0.025) | -0.019 (0.037) | 0.820 | 0.002 |
| Model 1 | Ref. | -0.012 (0.026) | -0.020 (0.038) | 0.727 | 0.042 |
| Total motility (%)^a^ |  |  |  |  |  |
| Crude model | Ref. | 0.001 (0.057) | -0.041 (0.082) | 0.871 | 0.001 |
| Model 1 | Ref. | 0.022 (0.058) | -0.022 (0.082) | 0.124 | 0.085 |
| Progressive motility (%) |  |  |  |  |  |
| Crude model | Ref. | 1.950 (2.614) | -3.017 (3.795) | 0.430 | 0.009 |
| Model 1 | Ref. | 3.462 (2.664) | -2.041 (3.809) | 0.081 | 0.092 |
| Normal sperm forms (%)^a^ |  |  |  |  |  |
| Crude model | Ref. | -0.016 (0.122) | 0.130 (0.175) | 0.708 | 0.004 |
| Model 1 | Ref. | -0.058 (0.126) | 0.127 (0.178) | 0.390 | 0.062 |

Values are B non-standardized coefficient and standard errors. ^a^Variable were natural log transformed to approximate to a normal distribution. ^b^Model *P-*value. Crude model: unadjusted. Model 1 adjusted for: plasma folate (nmol/L), plasma cobalamin (pmol/L), age (years), BMI (kg/m^2^), education (high school or less, college or high education), smoking status (non-smoker, current smoker, and former smoker), physical activity (MET min/week), abstinence days (days), and creatinine (µmol/L).

Abbreviations. %, percentage; BMI, body mass index; MET, metabolic equivalent of task; min, minute; MTRR, methionine transferase reductase; n, number of subjects.

**Supplementary Table S11.** Multiple linear regression analysis to test the associations between *MTRR* 66 A>G polymorphism and sperm quality parameters.

| **Sperm quality parameters** | **Categories of *MTRR* 66 A>G polymorphism** | | | | |
| --- | --- | --- | --- | --- | --- |
|  | **AA** | **AG vs. AA** | **GG vs. AA** | ***P-*value^b^** | **R^2^** |
| n | 56 | 86 | 52 |  |  |
| Total sperm count (x10^6^ spz.)^a^ |  |  |  |  |  |
| Crude model | Ref. | 0.072 (0.172) | 0.113 (0.193) | 0.835 | 0.002 |
| Model 1 | Ref. | 0.132 (0.170) | 0.184 (0.191) | 0.035 | 0.106 |
| Sperm concentration (x10^6^ spz./mL)^a^ |  |  |  |  |  |
| Crude model | Ref. | 0.081 (0.161) | 0.101 (0.181) | 0.831 | 0.002 |
| Model 1 | Ref. | 0.124 (0.163) | 0.145 (0.183) | 0.249 | 0.071 |
| Sperm vitality (%)^a^ |  |  |  |  |  |
| Crude model | Ref. | 0.046 (0.028) | -0.001 (0.031) | 0.147 | 0.02 |
| Model 1 | Ref. | 0.041 (0.029) | 0.002 (0.032) | 0.519 | 0.054 |
| Total motility (%)^a^ |  |  |  |  |  |
| Crude model | Ref. | 0.028 (0.063) | -0.003 (0.070) | 0.856 | 0.002 |
| Model 1 | Ref. | 0.043 (0.063) | 0.007 (0.071) | 0.128 | 0.084 |
| Progressive motility (%) |  |  |  |  |  |
| Crude model | Ref. | 0.276 (2.911) | -1.051 (3.265) | 0.902 | 0.001 |
| Model 1 | Ref. | 1.126 (2.928) | -0.419 (3.298) | 0.173 | 0.079 |
| Normal sperm forms (%)^a^ |  |  |  |  |  |
| Crude model | Ref. | 0.082 (0.134) | -0.095 (0.150) | 0.441 | 0.009 |
| Model 1 | Ref. | 0.091 (0.137) | -0.077 (0.153) | 0.343 | 0.065 |

Values are B non-standardized coefficient and standard errors. ^a^Variable were natural log transformed to approximate to a normal distribution. ^b^*P-*value < 0.05 for comparison of specific genotype versus normal homozygote genotype (reference). Crude model: unadjusted. Model 1 adjusted for: plasma folate (nmol/L), plasma cobalamin (pmol/L), age (years), BMI (kg/m^2^), education (high school or less, college or high education), smoking status (non-smoker, current smoker, and former smoker), physical activity (MET min/week), abstinence days (days), and creatinine (µmol/L).

Abbreviations. %, percentage; BMI, body mass index; MET, metabolic equivalent of task; min, minute; MTRR, methionine transferase reductase; n, number of subjects.

**Supplementary Table S12.** Multiple linear regression analysis to test the associations between *TCII* 776 C > G polymorphism and sperm quality parameters.

| **Sperm quality parameters** | **Categories of *TCII* 776 C > G polymorphism** | | | | |
| --- | --- | --- | --- | --- | --- |
|  | **CC** | **CG vs. CC** | **GG vs. CC** | ***P-*value** | **R^2^** |
| n | 64 | 88 | 43 |  |  |
| Total sperm count (x10^6^ spz.)^a^ |  |  |  |  |  |
| Crude model | Ref. | -0.028 (0.164) | -0.055 (0.197) | 0.961 | 0.001 |
| Model 1 | Ref. | -0.056 (0.165) | -0.077 (0.198) | 0.050 | 0.100 |
| Sperm concentration (x10^6^ spz./mL)^a^ |  |  |  |  |  |
| Crude model | Ref. | -0.111 (0.153) | -0.196 (0.184) | 0.552 | 0.006 |
| Model 1 | Ref. | -0.139 (0.158) | -0.213 (0.188) | 0.214 | 0.074 |
| Sperm vitality (%)^a^ |  |  |  |  |  |
| Crude model | Ref. | 0.038 (0.027) | 0.039 (0.032) | 0.304 | 0.012 |
| Model 1 | Ref. | 0.031 (0.028) | 0.029 (0.033) | 0.634 | 0.047 |
| Total motility (%)^a^ |  |  |  |  |  |
| Crude model | Ref. | -0.001 (0.059) | 0.131 (0.071) | 0.114 | 0.022 |
| Model 1 | Ref. | -0.029 (0.060) | 0.133 (0.072) | 0.022 | 0.113 |
| Progressive motility (%) |  |  |  |  |  |
| Crude model | Ref. | -2.268 (2.769) | 2.893 (3.323) | 0.254 | 0.014 |
| Model 1 | Ref. | -3.567 (2.814) | 2.976 (3.364) | 0.045 | 0.102 |
| Normal sperm forms (%)^a^ |  |  |  |  |  |
| Crude model | Ref. | -0.035 (0.130) | -0.072 (0.155) | 0.896 | 0.001 |
| Model 1 | Ref. | -0.080 (0.134) | -0.105 (0.159) | 0.431 | 0.059 |

Values are B non-standardized coefficient and standard errors. ^a^Variable were natural log transformed to approximate to a normal distribution. ^b^*P-*value < 0.05 for comparison of specific genotype versus normal homozygote genotype (reference). Crude model: unadjusted. Model 1 adjusted for: plasma folate (nmol/L), plasma cobalamin (pmol/L), age (years), BMI (kg/m^2^), education (high school or less, college or high education), smoking status (non-smoker, current smoker, and former smoker), physical activity (MET min/week), abstinence days (days), and creatinine (µmol/L).

Abbreviations. %, percentage; BMI, body mass index; MET, metabolic equivalent of task; min, minute; n, number of subjects; TCII, transcobalamin II.
